# Beyond efficacy: the treatment lifecycle of incretin and multi-receptor agonists

**DOI:** 10.3389/fendo.2026.1902185

**Published:** 2026-09-24

**Authors:** Syed Arman Rabbani, Mohamed El-Tanani, Ismail I. Matalka, Manfredi Rizzo

**Affiliations:** 1 RAK Medical and Health Sciences University, Ras Al Khaimah, United Arab Emirates; 2 School of Medicine, University of Palermo, Palermo, Italy

**Keywords:** cardiovascular, GLP-1 receptor agonist (RA), incretin, multi-receptor agonists, obesity, real-world, tirzepatide, type 2 diabetes

## Abstract

Incretin and multi-receptor agonist therapies have delivered the widest range of cardiometabolic benefit of any pharmacological class in a generation, with documented benefit across obesity, type 2 diabetes, cardiovascular and renal outcomes, heart failure with preserved ejection fraction, obstructive sleep apnea, and metabolic-dysfunction-associated steatohepatitis. A substantial fraction of the cardiovascular benefit is weight independent. The pivotal trial evidence is now mature, extending to the dedicated tirzepatide cardiovascular outcome program. The clinical and system-level processes that govern long-term cardiovascular and metabolic benefit, however, lie largely outside the efficacy envelope that these trials measure. Real-world persistence is approximately one-third at 12 months in non-diabetic obesity. Discontinuation is dominated by cost rather than therapeutic failure. Weight and cardiometabolic gains revert toward baseline within months of abrupt cessation. Cyclical use has emerged as a common real-world pattern among adults who discontinue and then restart therapy. Our review proposes a lifecycle framework that organizes the evidence along the patient journey from initiation through titration, on-treatment trajectory, adverse-event landscape, persistence, discontinuation, and reinitiation. Tirzepatide serves as the analytical center of gravity; the selective GLP-1 receptor agonist literature serves as the mature comparator. Seven analytical positions are advanced, and five specific trial designs are named as the research agenda the field requires, four of which remain unaddressed. The work ahead is to align trials, prescribing pathways, and access systems with the chronic-pharmacotherapy reality of the class.

## 1 Introduction

Incretin pharmacotherapy has expanded over two decades from a glucose-lowering strategy for type 2 diabetes into a class with documented benefit in obesity, atherosclerotic cardiovascular disease, heart failure with preserved ejection fraction, chronic kidney disease, obstructive sleep apnea, and metabolic-dysfunction-associated steatohepatitis (1–8). The dual GIP/GLP-1 receptor agonist tirzepatide (9) has raised the efficacy ceiling further: weight loss approaching the lower end of the range achievable with bariatric–metabolic surgery (10) is now attainable with a once-weekly injection, though absolute weight reductions with surgery remain larger.

Most contemporary reviews describe the field along the axis of efficacy; that framing is accurate but partial. It omits whether the gain trial participants achieved during months of supervised dose escalation, accommodation to gastrointestinal effects, and sustained injections can be reproduced in populations initiating therapy in routine practice. For most patients, the answer is no (11–13). First-year discontinuation is the modal outcome (11, 12). Lost weight is largely regained (14, 15), and cardiometabolic indices drift back toward baseline (14, 16, 17). The trial trajectory belongs, in practice, to the minority who tolerate titration, can afford the medication, secure a consistent supply, and continue therapy when the indication is chronic but the regimen feels finite (18–20).

Our review is built around that gap. The determinants of long-term benefit lie largely outside the efficacy envelope defined by trial endpoints. Initiation, titration, on-treatment durability, adverse events, persistence, discontinuation, and what happens at and after stopping describe a lifecycle along which benefit accrues, is sustained, or is forfeited. The shape of that lifecycle and the points at which clinical decisions preserve or relinquish accrued benefit have not been synthesized into a framework clinicians, trialists, and policymakers can use in common.

Tirzepatide serves as the analytical center of gravity for two reasons: it represents the current upper bound of class efficacy (21), and its lifecycle is still being defined, with the tirzepatide type 2 diabetes cardiovascular outcome trial SURPASS-CVOT reported in 2025 (138) and the obesity morbidity–mortality trial initiated but not reported (22). The selective GLP-1 receptor agonists (semaglutide in subcutaneous and oral formulations, liraglutide, and dulaglutide) provide the comparator literature and the mature evidence base. Where dual-agonist data are immature or absent, we treat the selective GLP-1 receptor agonist literature as a leading indicator.

The review covers adults with cardiometabolic indications. Pediatric and adolescent populations (23), pregnancy and lactation, and the bariatric-postoperative setting are acknowledged gaps where the evidence base is too thin for synthesis here.

Cardiometabolic medicine has been here before with rosiglitazone, muraglitazar, and torcetrapib; the incretin class has worked through its own regulatory-driven concerns: rodent thyroid C-cell tumors (24), pancreatitis (25), gallbladder disease (26), nonarteritic anterior ischemic optic neuropathy (27), and, most recently, suicidality (28). Each followed the familiar pattern of regulatory flag, epidemiological scrutiny, and partial or complete defusion (29, 30). The position taken here is neither defensive nor skeptical: the efficacy data are real and durable in those who continue therapy, the lifecycle problems are also real, and they grow more consequential as the efficacy ceiling rises. The analytical question organizing the sections that follow is what governs whether trial benefit accrues, is sustained, or is forfeited across the years a patient is meant to remain on therapy (**Figures 1**, **2**).

**Figure 1:**
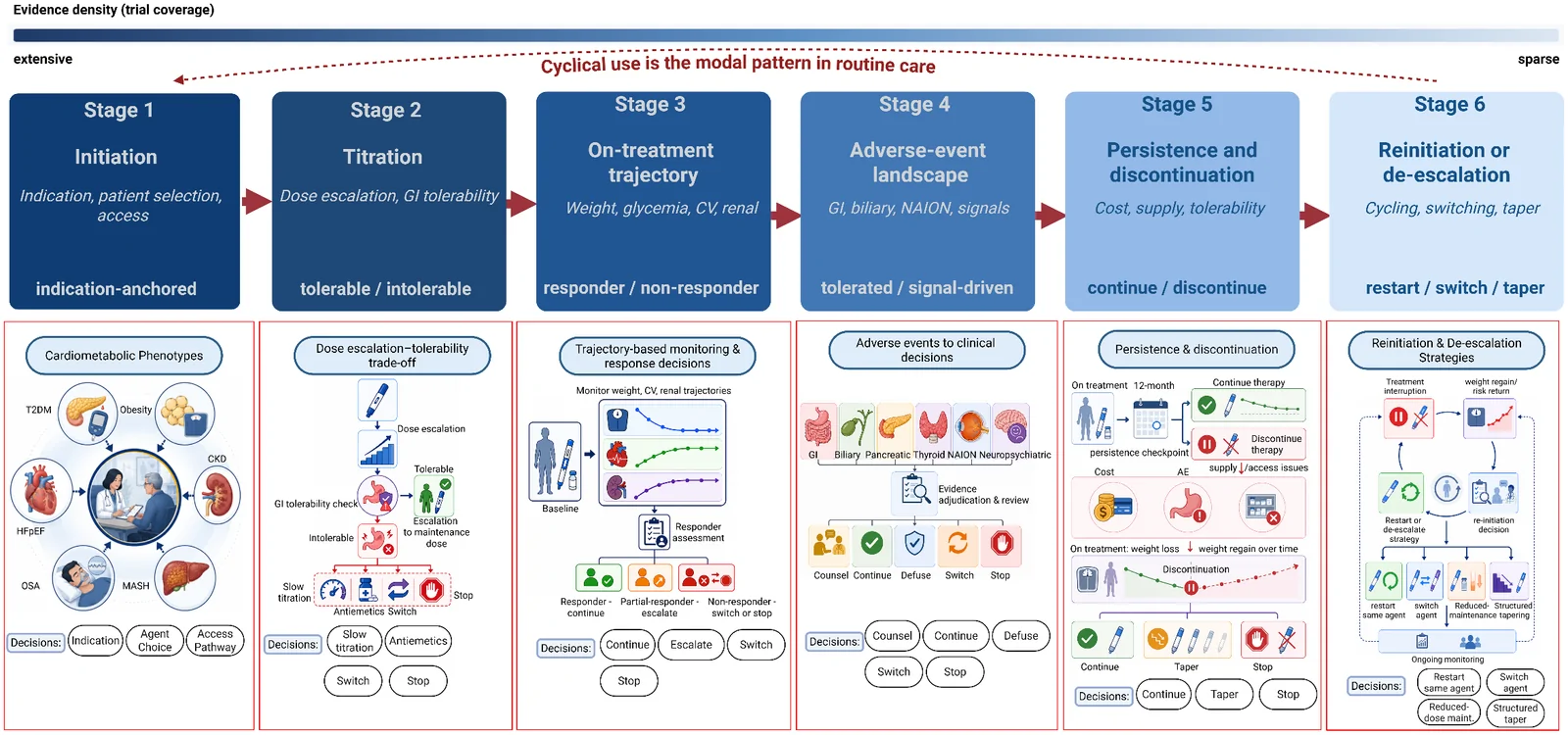
Lifecycle framework for incretin pharmacotherapy. Author-developed conceptual framework. Six-stage progression from initiation through reinitiation, with stage-specific clinical decisions in the lower row and the evidence-density gradient (extensive at stages 1–3 and sparser at stages 4–6) along the top. The dashed return arrow indicates that cyclical use, not permanent cessation, is the more common real-world pattern.

**Figure 2:**
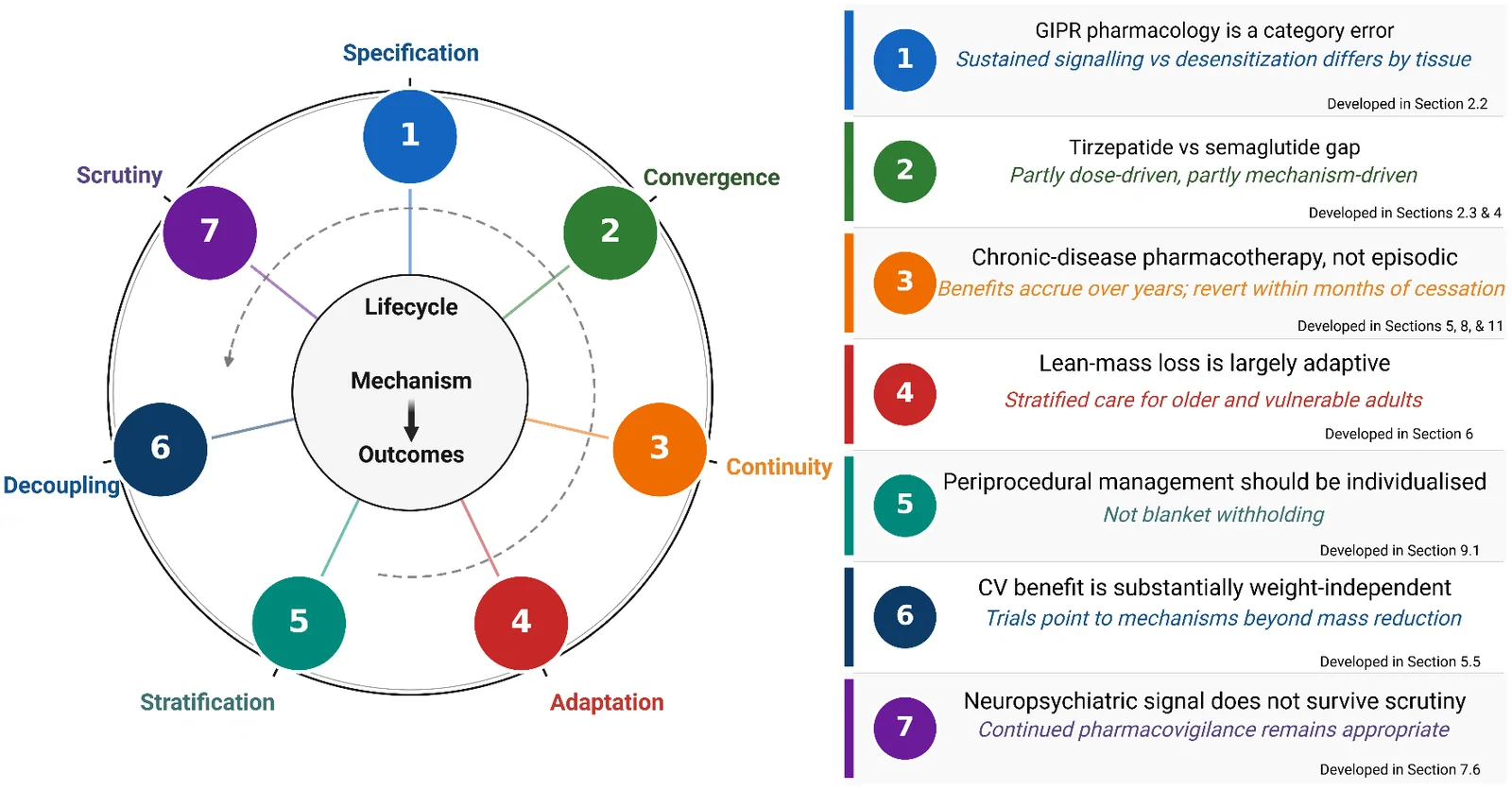
Seven analytical positions advanced in this review, mapped onto the incretin treatment lifecycle. This is an author-developed schema, not a consensus-derived diagram. The dial arranges the seven positions as stations around a lifecycle clock, with the dashed arc tracing the mechanism-to-outcome progression that organizes the review. Each position is paired with a single-noun conceptual reframing, namely—specification, convergence, continuity, adaptation, stratification, decoupling, scrutiny—naming the intellectual operation the position performs on the existing literature. The right-hand panel lists each numbered position with its full statement and the section in which it is developed.

This is a narrative and analytical review. The lifecycle framework proposed here and the seven analytical positions summarized in **Figure 2** are the authors’ syntheses of the primary literature rather than consensus statements from professional societies or expert panels and are flagged in text where interpretation extends beyond the direct evidence base. Evidence was prioritized by design hierarchy, giving greatest weight to randomized trials with pre-specified primary endpoints, then to adjudicated observational data, and then to unadjudicated real-world sources. Where estimates from trial and real-world sources diverged materially, the divergence is discussed in the relevant section rather than reconciled by preference for one source. The relevant literature was identified by searching PubMed/MEDLINE, Scopus, Cochrane Library, and ClinicalTrials.gov for English-language reports published up to April 30, 2026. The search terms included names of individual agents and pivotal trials together with terms covering incretin and multi-receptor pharmacology, the cardiometabolic indications, and the lifecycle stages addressed here. Representative search strings combined the drug and class terms (“semaglutide”, “tirzepatide”, “liraglutide”, “dulaglutide”, “exenatide”, “lixisenatide”, “retatrutide”, “survodutide”, “pemvidutide”, “cagrilintide”, “orforglipron”, “maridebart cafraglutide”, “GLP-1 receptor agonist”, “GIP receptor agonist”, “multi-receptor agonist”, “incretin”) with the indication and lifecycle terms (“type 2 diabetes”, “obesity”, “cardiovascular outcomes”, “renal outcomes”, “heart failure with preserved ejection fraction”, “obstructive sleep apnea”, “metabolic-dysfunction-associated steatohepatitis”, “MASH”, “titration”, “persistence”, “discontinuation”, “reinitiation”, “adverse events”, “periprocedural”, “pregnancy”, “adolescent”, “cost”, “access”). The inclusion criteria were peer-reviewed publications reporting studies in adults with cardiometabolic indications, ClinicalTrials.gov registry entries for late-phase trials, and regulatory documents from the FDA and EMA. The exclusion criteria were case reports, animal-only studies (except in mechanistic sections), non-English publications, and superseded conference abstracts where the peer-reviewed publication was available. The reference lists of key trials and recent reviews were screened for further sources. Priority was given to pivotal phase 3 randomized controlled trials, adjudicated cardiovascular and renal outcome trials, regulatory and pharmacovigilance safety analyses, and large real-world persistence and discontinuation cohorts; mechanistic and pharmacological studies were included where they informed clinical interpretation. Where dual-agonist data were immature or absent, the selective GLP-1 receptor agonist literature was treated as the comparator. Studies were selected for clinical relevance, methodological quality, and currency; the synthesis is intended to be balanced and evidence-based rather than exhaustive.

## 2 Pharmacology of incretin and multi-receptor agonism

### 2.1 Incretin system and selective GLP-1 receptor agonism

The two incretin hormones, glucagon-like peptide-1 (GLP-1) and glucose-dependent insulinotropic polypeptide (GIP), are released from L and K cells after enteral nutrient delivery and account for the larger insulin secretory response to oral compared with isoglycemic intravenous glucose (1, 31, 32). In type 2 diabetes, the incretin effect is markedly diminished: GIP retains plasma concentrations but loses much insulinotropic action with only partial recovery on glycemic correction (31, 33, 34), whereas GLP-1 retains insulin-stimulating activity. The differential drove the GLP-1 receptor (GLP-1R) into prominence as the pharmacological target through the 1990s–2000s, while the GIP receptor (GIPR) was treated as a problem of physiology rather than therapy (1).

The selective GLP-1R agonism clinical effects are well characterized: amplified glucose-dependent insulin secretion and suppression of inappropriate glucagon release from pancreatic alpha cells (1), reduced food intake and satiety via hypothalamic and brainstem signaling (1, 35), and slowed gastric and small intestinal motility, contributing to postprandial glucose flattening, appetite reduction, and most of the class’s GI adverse-event profile (36, 37). Weekly dosing was achieved through molecular engineering including fatty acid acylation and albumin binding, which extend the plasma half-life to about 1 week. The receptor’s relative resistance to *in vivo* desensitization supports the sustained pharmacodynamic effect under this dosing schedule rather than causing tachyphylaxis of the therapeutic response (1, 38).

The cardiovascular (CV) and renal benefits extend beyond what weight and glycemic improvement alone would predict—anti-inflammatory effects, blood pressure reduction, visceral adipose reduction, and direct renal hemodynamic actions all contribute—with the fractional partition between weight-mediated and weight-independent components contested (39–41) and adjudicated in the cardiovascular benefit mechanism discussion below.

### 2.2 GIPR pharmacology question

Tirzepatide engages both incretin receptors with asymmetric pharmacology. It produces approximately fivefold greater occupancy of GIPR than GLP-1R at therapeutic doses, and at GLP-1R it shows biased signaling favoring cyclic AMP generation over beta-arrestin recruitment with reduced receptor internalization (9, 38). Cryo-EM confirms the dual-engagement geometry (42).

The discussion accompanying tirzepatide’s development has often been framed as a binary choice between GIPR agonism and antagonism. The framing is partly a category error: The clinically relevant question is whether receptor engagement at a given tissue produces sustained signaling or desensitization, and the answer differs across three tissue compartments. At the pancreatic beta cell, agonism is acute and cooperative with GLP-1R; GIPR engagement amplifies glucose-dependent insulin secretion in a manner human islet studies confirm is required for the full insulin response observed with tirzepatide (43). In CN satiety circuits, agonism is sustained, with desensitization appearing slower or compartmentalized. Adipocyte exposure is chronic, and chronic agonism produces functional desensitization that pharmacologically resembles antagonism; peripheral GIPR-mediated mechanisms such as energy expenditure via futile calcium cycling appear to operate in this regime (44–46).

The maridebart cafraglutide program illustrates the same point from the other direction: A bispecific molecule combining GIPR antagonism with GLP-1R agonism produced clinically meaningful weight loss in early evaluation, with up to approximately 20% mean reduction at 52 weeks in the subsequent phase 2 trial (47, 139). The cleanest inference is not that GIPR antagonism is independently efficacious (no monotherapy trial has been conducted) but that similar endpoints can be reached from either direction along the GIPR dose–response curve because both pathways reduce productive GIPR signaling in the adipocyte while preserving GLP-1R-mediated effects at the beta cell [which require GLP-1R agonism rather than GIPR agonism, per (43)] and at CNS satiety circuits. The agonism–antagonism debate has matured beyond a categorical answer; the operative pharmacology should be specified at the tissue and timescale, not the receptor (**Figure 3**).

**Figure 3:**
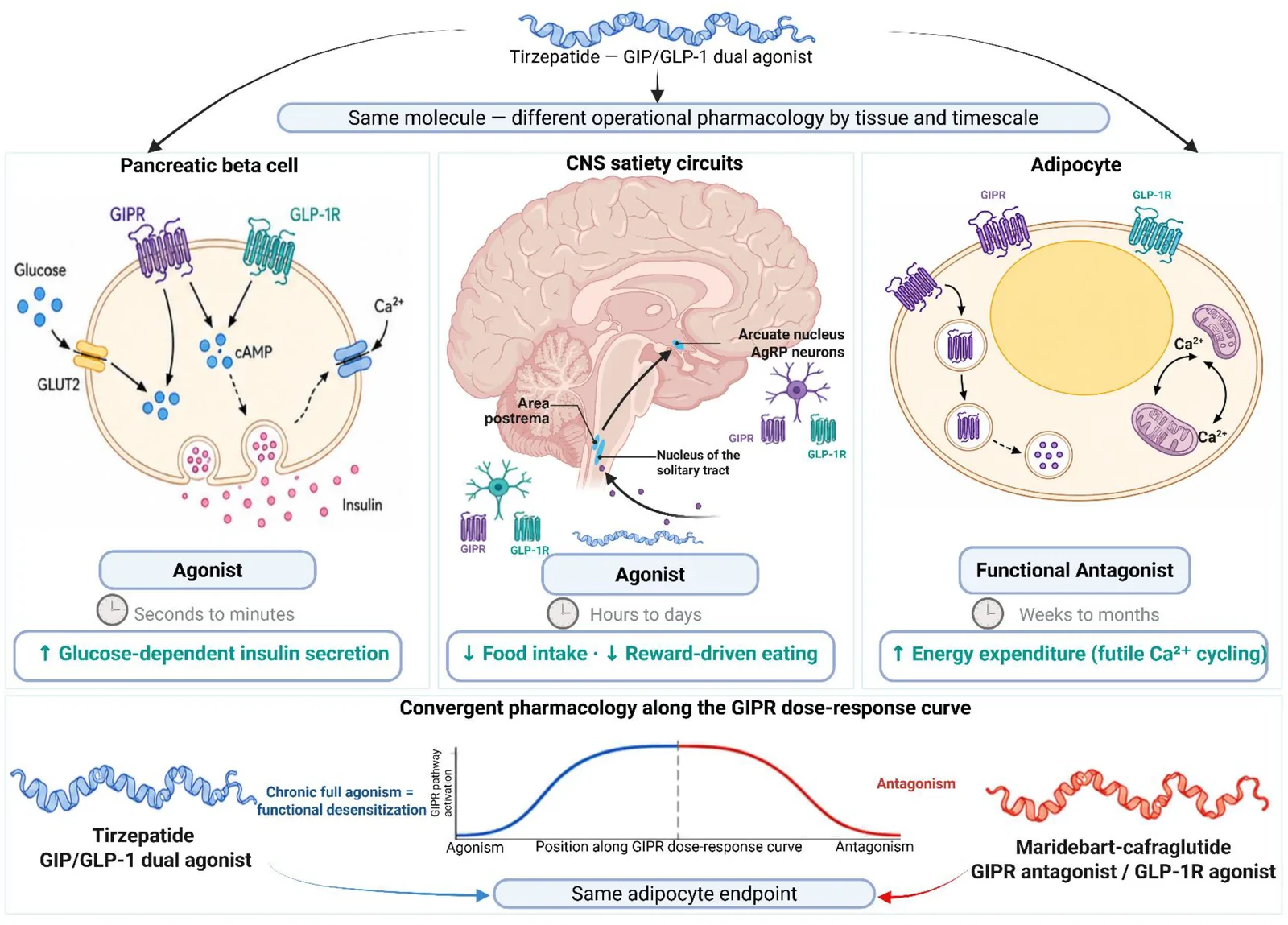
Tissue-level GIPR pharmacology: the same molecule, different operational pharmacology by tissue and timescale. Working-model schematic showing how tirzepatide is proposed to engage GIPR across three tissue compartments to produce different operational states. The adipocyte functional-antagonism panel and the maridebart cafraglutide convergence panel below rest primarily on preclinical rodent and adipocyte data, and their extension to human tissue is inferred rather than demonstrated. Pancreatic beta cell (left): Acute, cooperative agonism with GLP-1R amplifies glucose-dependent insulin secretion via cAMP; operational state, agonist; timescale, seconds to minutes. CNS satiety circuits (middle): Sustained agonism in the arcuate nucleus and area postrema reduces food intake and reward-driven eating, with slow or compartmentalized desensitization; operational state, agonist; timescale, hours to days. Adipocyte (right): Chronic exposure produces receptor internalization and functional desensitization, allowing peripheral GIPR-mediated energy expenditure via futile calcium cycling; operational state, functional antagonist; timescale, weeks to months. Bottom convergence panel: Maridebart cafraglutide (GIPR antagonist/GLP-1R agonist) reaches the same reduced-productive-adipocyte-signaling endpoint from the opposite direction along the GIPR pharmacology spectrum (45, 46). The figure visualizes the central claim that the agonism-versus-antagonism debate is partly a category error. The operative pharmacology should be specified at the tissue and timescale, not the receptor.

### 2.3 Multi-receptor agonism and the basis of differential efficacy

Dual agonism’s structural and signaling basis explains some but not all of the clinical-efficacy difference between tirzepatide and selective GLP-1R agonists. Cryo-EM data establish that a single peptide can engage both receptors with appropriate conformational selectivity (42), and next-generation multi-receptor agents extend the principle to the glucagon receptor, adding an energy-expenditure component (38, 48, 49). Tirzepatide’s half-life of approximately 5 days supports weekly dosing, and its gastric-emptying effect develops tachyphylaxis over the first 2 to 12 weeks of treatment (36, 50). These features bear directly on the titration window discussed below, periprocedural management of retained gastric contents, and concomitant oral medication handling (37).

Why tirzepatide produces greater weight loss than semaglutide in head-to-head comparison (21) has been answered two ways, neither of which is sufficient alone. The first invokes dose-equivalence: SURMOUNT-5 compared maximum-tolerated tirzepatide (10 or 15 mg weekly) against the maximum approved semaglutide for obesity (2.4 mg weekly), and STEP UP subsequently established that 7.2 mg semaglutide (18.7% weight loss, treatment-policy estimand, efficacy estimand 20.7%) narrows but does not close the differential (51). The second invokes mechanism: GIPR agonism contributes to insulin sensitization and to cardiometabolic biomarker change in ways not explained by weight loss alone, which GLP-1R monotherapy at any dose appears unable to fully reproduce (39, 52, 53). The most defensible reading is that the difference is partly dose-driven and partly mechanism-driven, with the relative contribution varying across populations. In non-diabetic obesity, dose equivalence likely accounts for a larger share. In type 2 diabetes, where GIPR engagement at the beta cell does mechanistic work GLP-1R agonism at any dose cannot substitute for, the mechanism-driven component is larger (43, 52, 53). Tolerability was broadly similar between agents in SURMOUNT-5 but greater at semaglutide 7.2 mg than at 2.4 mg in STEP UP (21, 51).

## 3 Patient selection across cardiometabolic phenotypes

### 3.1 Type 2 diabetes

SURPASS established tirzepatide superior to placebo in drug-naive T2D (54), titrated insulin degludec (55), titrated insulin glargine at increased cardiovascular risk (56), and semaglutide 1 mg in head-to-head comparison (57). Mean HbA1c reductions of 1.7 to 2.6 percentage points and weight reductions of 5.4 to 11.7 kg were achieved at the higher tirzepatide doses, with cardiovascular safety meeting conventional thresholds in pooled adjudicated analyses (58), pending dedicated outcome trials in T2D [SURPASS-CVOT, reported in 2025, showed non-inferiority of tirzepatide against dulaglutide for 3-point MACE (HR 0.92) but did not reach superiority (138)] and obesity [SURMOUNT-MMO (22)].

For selective GLP-1 receptor agonists, the cardiovascular and renal evidence in type 2 diabetes is mature. LEADER (59), SUSTAIN-6 (60), REWIND (61), and AMPLITUDE-O (62) each reduced MACE across established CVD and lower-risk primary prevention. FLOW (7) reduced the composite renal endpoint by 24% and cardiovascular death by 29% in type 2 diabetes with chronic kidney disease, and SOUL (5) extended CV benefit to oral semaglutide. Pooled across the class, MACE reduction is approximately 12% to 14%, and renal composite reduction is approximately 17% to 21% (63, 64). The differential among agents [principally exendin-based without MACE benefit: ELIXA (65), EXSCEL (66)] and native-GLP-1 analogues that did (LEADER, SUSTAIN-6, REWIND, SOUL) reflects pharmacokinetics and signaling differences not yet formally adjudicated within the class.

### 3.2 Obesity, with and without type 2 diabetes

SURMOUNT-1 produced approximately 21% weight reduction at 72 weeks with tirzepatide 15 mg (2), sustained at 3 years with a markedly reduced incidence of new-onset type 2 diabetes (67). The equivalent figure for semaglutide 2.4 mg in STEP-1 is approximately 15% at 68 weeks (68), and SURMOUNT-5 confirmed tirzepatide’s superiority head to head (21). The historical anchor is SCALE Obesity with liraglutide (69), which produced approximately 8% weight reduction at 56 weeks.

A diabetes-status modifier holds across both programs: Weight loss with tirzepatide is approximately 6 percentage points lower in SURMOUNT-2 (obesity with T2D) than in SURMOUNT-1 (obesity without diabetes) (2, 70); the analogous comparison between STEP-2 and STEP-1 produces a similar attenuation (68, 71). The mechanisms are incompletely characterized, but the reproducibility of the effect should temper expectation when initiating these agents in adults with both obesity and type 2 diabetes.

### 3.3 Cardiometabolic phenotypes beyond diabetes and obesity

In obesity-related HFpEF, the STEP-HFpEF program established that semaglutide improves symptoms, function, and quality of life (72–74), and SUMMIT demonstrated that tirzepatide reduces the composite of cardiovascular death or worsening heart-failure events (HR 0.62, 95% CI 0.41 to 0.95) (75), with secondary analyses showing reductions in systolic blood pressure, estimated blood volume, C-reactive protein, and troponin T suggesting structural cardiorenal benefit beyond weight loss (3). SELECT identified benefit across HFpEF and HFrEF in non-diabetic obesity with prior cardiovascular disease (76).

Obstructive sleep apnea with obesity is a treatable target. SURMOUNT-OSA produced clinically meaningful apnea–hypopnea index reduction at 52 weeks (4), with mediation analysis indicating an OSA-specific contribution to cardiometabolic improvement beyond weight loss (77). The earlier SCALE Sleep Apnea trial of liraglutide identified a smaller but directionally consistent signal 8 years before (78). The durability question is sharper than the efficacy question: AHI improvement at 52 weeks does not establish whether benefit persists over years of chronic OSA management or translates to the cardiovascular events associated with comorbid OSA.

In metabolic-dysfunction-associated steatohepatitis, SYNERGY-NASH, a phase 2 trial, established tirzepatide’s effect on histological resolution and fibrosis at 52 weeks (8). The ESSENCE phase 3 program for semaglutide 2.4 mg reported at its 72-week interim analysis that 62.9% of the participants achieved resolution of steatohepatitis without worsening fibrosis versus 34.3% on placebo, and 36.8% achieved improvement in fibrosis without worsening steatohepatitis versus 22.4% (141). On the basis of these data, the US FDA granted accelerated approval in August 2025 for semaglutide 2.4 mg in adults with non-cirrhotic MASH and F2 to F3 fibrosis (FDA regulatory news release) (142). Semaglutide is therefore currently the incretin agent with the most advanced hepatic-outcome evidence, and the earlier phase 2 semaglutide data (79, 80) should be read in light of the subsequent phase 3 confirmation. Retatrutide (81), survodutide (82), and pemvidutide (83) extend hepatic benefit to triple agonism, glucagon co-agonism, and the related multi-receptor space. The principal non-incretin comparator is resmetirom, a thyroid-hormone-receptor-beta agonist (84). The durability question is incompletely addressed at the trial level: histological endpoints anchor at 52 weeks while MASH unfolds over decades, and no dedicated RCT has yet linked histological improvement to clinical hepatic events (cirrhosis decompensation, hepatocellular carcinoma).

Renal outcomes are among the most quantitatively secure non-glycemic domains. FLOW remains the defining kidney outcome trial for semaglutide in type 2 diabetes with chronic kidney disease (7), while SELECT renal analyses extend kidney-outcome benefit to non-diabetic obesity with CVD (86). SOUL confirms cardiovascular benefit with oral semaglutide in high-risk type 2 diabetes with ASCVD, CKD, or both but did not show a statistically significant reduction in its confirmatory major kidney disease endpoint (5, 85). The FLOW SGLT2 inhibitor subgroup analysis (87) indicates that renal benefit is preserved in patients on baseline SGLT2 inhibitor therapy.

### 3.4 Positioning relative to SGLT2 inhibitors and metabolic surgery

The class structure has stabilized around two pillars. SGLT2 inhibitors deliver renal and cardiovascular benefit principally through hemodynamic and natriuretic mechanisms, with a modest effect on weight. Incretin and multi-receptor agonists deliver substantial effects on weight and benefits across the cardiometabolic phenotypes outlined above. The two classes can be used together with preserved efficacy and additive benefit on several endpoints, and current guidelines (88–93, 144, 145) increasingly recommend combination therapy in patients with multiple indications.

Metabolic surgery retains a substantial weight-reduction advantage (approximately 22 kg greater in randomized data), with similar glycemic outcomes and a possible advantage for heart failure prevention through weight-loss-independent mechanisms (10, 94). The SURMOUNT and SURMOUNT-OSA results have placed a once-weekly injection within an efficacy range previously exclusive to bariatric–metabolic surgery. Contemporary agents narrow, but do not close, the gap. The sequencing question (pharmacotherapy first or surgery first, transition between modalities, management of weight regain after either) is among the most clinically consequential questions the field has yet to answer. **Table 1** presents the pivotal trial landscape for incretin and multi-receptor agonists.

**Table 1:** Pivotal trial landscape for incretin and multi-receptor agonists.

Trial	Population	Intervention	Comparator	Follow-up	Primary endpoint	Key result
Type 2 diabetes-selective GLP-1 receptor agonist cardiovascular outcome trials						
ELIXA (65)	Type 2 diabetes with acute coronary syndrome	Lixisenatide 10–20 mcg daily	Placebo	Median 25 months	4-point MACE (CV death, MI, stroke, hospitalization for unstable angina)	HR 1.02 (95% CI 0.89–1.17); non-inferior, no superiority
LEADER (59)	Type 2 diabetes at high cardiovascular risk	Liraglutide 1.8 mg daily	Placebo	Median 3.8 years	3-point MACE	HR 0.87 (95% CI 0.78–0.97); 13% relative risk reduction
SUSTAIN-6 (60)	Type 2 diabetes with established CVD/CKD or 60+ years with risk factors	Semaglutide 0.5 or 1.0 mg weekly	Placebo	Median 2.1 years	3-point MACE	HR 0.74 (95% CI 0.58–0.95); 26% relative risk reduction
EXSCEL (66)	Type 2 diabetes with or without prior CVD	Exenatide once weekly	Placebo	Median 3.2 years	3-point MACE	HR 0.91 (95% CI 0.83–1.00); non-inferior, superiority *P* = 0.06
REWIND (61)	Type 2 diabetes (32% with prior CVD; primary-prevention-enriched)	Dulaglutide 1.5 mg weekly	Placebo	Median 5.4 years	3-point MACE	HR 0.88 (95% CI 0.79–0.99); 12% relative risk reduction
AMPLITUDE-O (62)	Type 2 diabetes with CVD or CKD	Efpeglenatide 4 or 6 mg weekly	Placebo	Median 1.8 years	3-point MACE	HR 0.73 (95% CI 0.58–0.92); 27% relative risk reduction
SOUL (5)	Type 2 diabetes with established ASCVD or CKD	Oral semaglutide 14 mg daily	Placebo	Median 49.5 months	3-point MACE	HR 0.86 (95% CI 0.77–0.96); 14% relative risk reduction
Type 2 diabetes – Tirzepatide (SURPASS program)						
SURPASS-1 (54)	Type 2 diabetes inadequately controlled with diet and exercise alone	Tirzepatide 5, 10, or 15 mg weekly	Placebo	40 weeks	HbA1c change from baseline	–1.87% to –2.07% absolute reduction across doses; weight –7.0 to –9.5 kg
SURPASS-2 (57)	Type 2 diabetes on metformin	Tirzepatide 5, 10, or 15 mg weekly	Semaglutide 1 mg weekly	40 weeks	HbA1c change from baseline	Tirzepatide superior at all doses (ETD –0.15 to –0.45%); weight –1.9 to –5.5 kg vs. semaglutide
SURPASS-3 (55)	Type 2 diabetes on metformin with or without SGLT2 inhibitor	Tirzepatide 5, 10, or 15 mg weekly	Titrated insulin degludec	52 weeks	HbA1c change from baseline	Tirzepatide superior to degludec (ETD –0.59 to –1.04%); weight –7.5 to –12.9 kg
SURPASS-4 (56)	Type 2 diabetes, increased cardiovascular risk	Tirzepatide 5, 10, or 15 mg weekly	Titrated insulin glargine	52 weeks	HbA1c change from baseline	Tirzepatide superior to glargine; MACE-4 incidence numerically lower
SURPASS-CVOT (138)	Type 2 diabetes with established ASCVD	Tirzepatide up to 15 mg weekly	Dulaglutide 1.5 mg weekly (active comparator)	Median 4 years	3-point MACE	12.2% vs. 13.1% (HR 0.92); non-inferiority *P* = 0.003, superiority *P* = 0.09. Noninferior but not superior for 3-point MACE; tirzepatide produced greater metabolic improvements, but mechanistic contribution requires additional analyses
Obesity-selective GLP-1 receptor agonists						
SCALE Obesity (69)	Obesity without diabetes	Liraglutide 3.0 mg daily	Placebo	56 weeks	% body weight change	–8.0% vs. –2.6% placebo
STEP-1 (68)	Obesity/overweight with comorbidity, no type 2 diabetes	Semaglutide 2.4 mg weekly	Placebo	68 weeks	% body weight change	–14.9% vs. –2.4% placebo
STEP-2 (71)	Obesity with type 2 diabetes	Semaglutide 2.4 mg or 1.0 mg weekly	Placebo	68 weeks	% body weight change	–9.6% (2.4 mg) vs. –3.4% placebo
STEP-4 (118)	Obesity, semaglutide responders after 20-week open-label lead-in	Continue semaglutide 2.4 mg weekly	Switch to placebo	48 weeks after randomization	% body weight change from randomization	–7.9% continued vs. +6.9% placebo (analogue to SURMOUNT-4)
STEP-5 (99)	Obesity without type 2 diabetes	Semaglutide 2.4 mg weekly	Placebo	104 weeks (2 years)	% body weight change at 104 weeks	–15.2% vs. –2.6% placebo; durability at two years
STEP UP (Wharton 2025) (51)	Obesity without type 2 diabetes	Semaglutide 7.2 mg weekly	Semaglutide 2.4 mg or placebo	72 weeks	% body weight change	–18.7% (7.2 mg) vs. –15.6% (2.4 mg) vs. –3.9% placebo; ~half achieved at least 20% loss; narrows but does not close the tirzepatide differential
Obesity—tirzepatide (SURMOUNT program)						
SURMOUNT-1 (2)	Obesity, no diabetes	Tirzepatide 5, 10, or 15 mg weekly	Placebo	72 weeks	% body weight change	–15.0%/–19.5%/–20.9% across doses vs. –3.1% placebo
SURMOUNT-2 (70)	Obesity with type 2 diabetes	Tirzepatide 10 or 15 mg weekly	Placebo	72 weeks	% body weight change	–12.8%/–14.7% vs. –3.2% placebo
SURMOUNT-4 (16)	Obesity, randomized withdrawal	Tirzepatide maximum tolerated dose (continued) after 36-week open-label lead-in	Switch to placebo	52 weeks after randomization	% body weight change from randomization	–5.5% continued tirzepatide vs. +14.0% placebo; 89.5% vs. 16.6% maintained at least 80% of lead-in loss
SURMOUNT-5 (21)	Obesity, no diabetes	Tirzepatide maximum tolerated dose (10 or 15 mg)	Semaglutide max tolerated dose (1.7 or 2.4 mg)	72 weeks	% body weight change	–20.2% vs. –13.7% (head-to-head)
SURMOUNT-1 3-year extension (67)	Obesity with prediabetes (SURMOUNT-1 substudy)	Tirzepatide 5, 10, or 15 mg weekly	Placebo	176 weeks + 17-week off-treatment	% body weight change and progression to type 2 diabetes	Weight –12.3%/–18.7%/–19.7% across doses vs. –1.3% placebo at 176 weeks; HR 0.07 for incident T2D during treatment (94% RRR); HR 0.12 (95% CI 0.1–0.2) after 17-week off-treatment
Obesity-related cardiometabolic complications: heart failure, sleep apnea, steatohepatitis, cardiovascular and renal outcomes						
STEP-HFpEF (72)	Obesity-related HFpEF, non-diabetic; *n* = 529	Semaglutide 2.4 mg weekly	Placebo	52 weeks	Dual primary: KCCQ-CSS change and % body weight change	KCCQ-CSS +16.6 vs. +8.7 points (estimated difference +7.8 points, 95% CI 4.8–10.9); weight –13.3% vs. –2.6% placebo
SUMMIT (75); secondary analysis (3)	Obesity with HFpEF; *n* = 731 (364 tirzepatide/367 placebo)	Tirzepatide up to 15 mg weekly	Placebo	Median 2 years (primary trial); secondary analyses additional	Primary: composite of CV death or worsening HF event. Secondary: cardiometabolic (BP, blood volume, CRP, troponin T)	Primary endpoint HR 0.62 (95% CI 0.41–0.95) for CV death/worsening HF (38% RRR) (75); secondary analysis showed reduced systolic BP, estimated blood volume, hsCRP, troponin T (structural cardiorenal benefit) (3)
SURMOUNT-OSA (4)	Moderate to severe obstructive sleep apnea with obesity	Tirzepatide 10 or 15 mg weekly	Placebo	52 weeks	Apnea–hypopnea index change	–25.3 to –29.3 events per hour vs. –5.3 to –5.5 placebo (ETD –20.0 to –23.8)
SCALE Sleep Apnea (78)	Obesity with moderate–severe obstructive sleep apnea	Liraglutide 3.0 mg daily	Placebo	32 weeks	Apnea–hypopnea index change	–12.2 vs. –6.1 events per hour (ETD –6.1); precursor to SURMOUNT-OSA
SYNERGY-NASH (8)	MASH with significant fibrosis (F2–F3), majority with type 2 diabetes	Tirzepatide 5, 10, or 15 mg weekly	Placebo	52 weeks	MASH resolution without worsening of fibrosis	44–62% across tirzepatide doses vs. 10% placebo
SELECT (incl. renal substudy and HF subgroup) (6, 76, 86, 100)	Obesity, prior cardiovascular disease, non-diabetic, *n* = 17,604	Semaglutide 2.4 mg weekly	Placebo	Median 39.8 months	3-point MACE; renal substudy; HF subgroup	MACE HR 0.80 (95% CI 0.72–0.90); 20% RRR. Renal benefit confirmed; HF subgroup benefit across HFpEF and HFrEF phenotypes
FLOW (incl. SGLT2 inhibitor subgroup) (7, 87)	Type 2 diabetes with chronic kidney disease	Semaglutide 1.0 mg weekly	Placebo	Median 3.4 years	Composite kidney outcome	HR 0.76 (95% CI 0.66–0.88); 24% RRR; renal benefit preserved in patients on baseline SGLT2 inhibitor
SCALE 3-year extension (le Roux 2017) (101)	Obesity with prediabetes (BMI ≥30 or ≥27 with comorbidities); *n* = 2,254 randomized 2:1	Liraglutide 3.0 mg daily	Placebo	160 weeks	Time to onset of type 2 diabetes	HR 0.21 (95% CI 0.13–0.34) for incident T2D; 2% liraglutide vs. 6% placebo developed T2D at 160 weeks; weight –6.1% vs. –1.9% (estimated treatment difference –4.3%)
Pipeline—phase 2/3 trials in late-stage development						
Retatrutide phase 2 (Jastreboff 2023) (48)	Obesity without type 2 diabetes	Retatrutide (triple GIP/GLP-1/glucagon receptor agonist) 1–12 mg weekly	Placebo	48 weeks	% body weight change	–24.2% at 12 mg dose vs. –2.1% placebo; highest weight loss reported in a controlled trial
ATTAIN-1 (orforglipron phase 3) (129)	Obesity or overweight with comorbidity, without type 2 diabetes	Orforglipron (oral non-peptide GLP-1 receptor agonist) 6, 12, or 36 mg daily	Placebo (3:3:3:4 randomization)	72 weeks	% body weight change	At 36 mg: –11.2% vs. –2.1% placebo (treatment-regimen estimand, primary endpoint); –12.4% vs. –0.9% (efficacy estimand); 59.6% achieved at least 10% loss, 39.6% at least 15% (efficacy estimand)
REDEFINE-1 (CagriSema phase 3a) (133)	Adults without type 2 diabetes with at least one weight-related comorbidity; *n* = 3,417	CagriSema (cagrilintide 2.4 mg + semaglutide 2.4 mg) weekly; 4-arm design also testing cagrilintide alone and semaglutide alone	Placebo	68 weeks	% body weight change	CagriSema –20.4% vs. semaglutide –14.9% vs. cagrilintide –11.5% vs. placebo –3.0%; 60% of CagriSema participants achieved at least 20% loss, 23% at least 30%; approaches tirzepatide efficacy range
Survodutide phase 2 MASH (Sanyal 2024) (82)	MASH with biopsy-confirmed fibrosis stage F1–F3	Survodutide (glucagon/GLP-1 dual receptor agonist) 2.4, 4.8, or 6.0 mg weekly	Placebo	48 weeks (24-week titration + 24-week maintenance)	MASH improvement without worsening of fibrosis	MASH improvement: 47% (2.4 mg), 62% (4.8 mg), 43% (6.0 mg) vs. 14% placebo; ≥30% liver fat reduction in 57%–67% vs. 14% placebo; fibrosis improvement ≥1 stage in 34%–36% vs. 22% placebo
OASIS-1 (132)	Obesity	Oral semaglutide 50 mg daily	Placebo	68 weeks	% body weight change	–15.1% vs. –2.4% placebo; oral dosing reaches phase 3 injectable efficacy range
Maridebart cafraglutide (MariTide) phase 2 (47, 139)	Two cohorts (*n* = 592 total): cohort A, obesity/overweight without type 2 diabetes (*n* = 465); cohort B, obesity/overweight with type 2 diabetes (*n* = 127)	Maridebart cafraglutide (GIPR antagonist + GLP-1R agonist bispecific peptide-antibody conjugate); monthly Q4W subcutaneous dosing	Placebo	52 weeks	% body weight change (both cohorts); HbA1c (cohort B)	Cohort A: −12.3% to −16.2% across doses vs. −2.5% placebo (treatment policy estimand, primary endpoint); −16.3% to −19.9% under efficacy estimand. Cohort B: −8.4% to −12.3% vs. −1.7% placebo (treatment policy estimand); −12.1% to −17.0% under efficacy estimand; HbA1c −1.2 to −1.6 pp vs. +0.1 pp placebo (up to −2.2% under efficacy estimand). Weight loss had not plateaued at 52 weeks. Phase 1 (47); phase 2 (139); phase 3 MARITIME programme initiated

## 4 Initiation, titration, and the tolerability trade-off

### 4.1 Dose escalation and the gastrointestinal burden

The GI adverse-event profile is both dose and titration dependent. Nausea, vomiting, diarrhea, and constipation cluster during the first 16–20 weeks of treatment and attenuate substantially as gastric-emptying tachyphylaxis develops (36, 50). In the pooled SURMOUNT-1 to SURMOUNT-4 analysis, gastrointestinal events were reported by approximately 28% to 73% of tirzepatide-treated participants depending on dose and population (95). The mediation analysis from that program is informative beyond the rate question: nausea, vomiting, and diarrhea together explained approximately 3% of total weight loss, which refutes the argument that weight reduction is largely an artefact of gastrointestinal intolerance (95).

Head-to-head trials in which gastrointestinal events were prespecified secondary safety endpoints [SURPASS-2 (57) and SURMOUNT-5 (21)] report modestly higher tirzepatide gastrointestinal event rates at maximum doses versus semaglutide, which was most pronounced during titration and at 15 mg. Strategies keeping otherwise intolerant patients on a path to maintenance include slowing the 4-week dose interval, smaller and slower-eaten meals, and short-course antiemetic therapy. Titration scheduling itself determines whether a patient stays on therapy long enough to benefit. Trial-defined schedules escalate every 4 weeks to maintenance dose at weeks 16 to 20. Slowing the schedule is a reasonable response to tolerability problems but extends the period during which the patient is paying for therapy that has not reached its effect.

### 4.2 Real-world titration falls short of the trial schedule

Pivotal-trial titration schedules are not the schedules patients receive in routine care. A UK primary-care cohort of 8,200 adults initiating GLP-1 receptor agonist therapy between 2018 and 2023 found that only 58% of subcutaneous/oral semaglutide initiators reached the recommended maintenance dose, and 21% remained on the starting dose by the second prescription (19). Mechanisms are heterogeneous: prescriber caution, patient request, supply interruption, and lack of follow-up.

Cost and insurance-related issues are the strongest documented barrier to treatment discontinuation and plausibly contribute to incomplete titration. Patients commonly remain on lower doses because of insurance step-therapy requirements that defer the higher dose since the lower-dose pen is the only one their plan covers or because the patient cannot absorb the out-of-pocket cost of uptitration (18, 20). The patient still on the starting dose in the first treatment year is rarely there for pharmacological reasons; the system has failed the titration.

The consequences are substantial. Real-world weight loss across electronic health record and claims cohorts averages 8% to 9% at 1 year for adults initiating semaglutide or tirzepatide for obesity compared with approximately 15% in STEP-1 and 21% in SURMOUNT-1 (13, 19, 96). Most of the attenuation is explained by early discontinuation and failure to reach maintenance dose (13, 19). Multidisciplinary obesity-clinic cohorts actively managing titration and addressing cost achieve weight loss closer to phase 3 figures (96), which suggests that the implementation gap is amenable to structural rather than pharmacological intervention.

### 4.3 Non-responder phenotype

A clinically meaningful minority fail to achieve 5% weight reduction despite reaching maintenance dose and continuing 6 months or longer. The proportion is consistently in the range of 10%–15% in non-diabetic obesity and higher in adults with concurrent type 2 diabetes (2, 68, 70, 96). None of the decisions about when to escalate, switch, or discontinue is supported by high-quality evidence.

Early response is the strongest available predictor, although late-response phenotypes are being characterized in the extension analyses of STEP-5 and SURMOUNT-1 in which a fraction of the participants who did not meet the 5% threshold at 6 months converted to responders by 12 to 18 months (67, 99). This late-response signal argues against strict early stopping rules pending prospective validation. The SCALE Teens analysis identified that adolescents who had not achieved 4% BMI reduction at 16 weeks of liraglutide were unlikely to reach 5% by trial end (97), and analogous patterns have been described in adult cohorts for both semaglutide and tirzepatide (13, 96). Whether early response should be used as a structured stopping rule is an open question for clinical and access reasons. Stopping at 16 weeks without 5% weight loss may be premature, may be cost-driven, or may be exactly what the lifecycle frame would recommend, and the literature does not yet adjudicate among the three.

Switching is the most common response, dominated by semaglutide to tirzepatide (11, 96, 98). The pharmacological premise of that switch has not been tested formally in patients who have failed on semaglutide; the trial-derived superiority of tirzepatide (21) was established in agent-naive populations. The prevailing switching practice extrapolates from agent-naive populations to a patient phenotype the trial literature has not addressed. Clinical observation suggests that tirzepatide retains efficacy after semaglutide, but whether the within-class superiority magnitude is preserved, is attenuated, or differs by failure phenotype requires the agent-switching trial named in the research agenda below.

## 5 On-treatment trajectory and durability of benefit

### 5.1 Weight and glycemic trajectory

Weight loss with incretin therapy follows a characteristic trajectory: a steep initial phase coincident with titration, slowing from approximately month 9, with a plateau between months 12 and 18 (2, 68). The plateau weight depends on agent, dose, and indication—semaglutide at 2.4 mg averages ~15% reduction in non-diabetic obesity (68) and tirzepatide at 15 mg to ~21% (2)—and the difference persists at extended follow-up (67, 99, 100). STEP-5 (99), SELECT 208-week extension (100), and SURMOUNT-1 3-year extension (67) established durability with continued therapy.

The T2D glycemic trajectory is similar: HbA1c reduction is rapid (targets within 8–12 weeks for many) and sustained through SURPASS (54–57) and SUSTAIN programs. In populations without diabetes, the glycemic effect emerges as delay or prevention of incident type 2 diabetes—the SURMOUNT-1 3-year extension demonstrated 88% lower hazard of progression from prediabetes (67), and the SCALE 3-year extension showed a hazard ratio of 0.21 for incident diabetes over 160 weeks of liraglutide (101). Disease prevention persists only while therapy continues.

### 5.2 Cardiovascular and renal trajectory

Both cardiovascular and renal trajectories are accrual dependent. The pivotal CVOTs in type 2 diabetes (LEADER (59), SUSTAIN-6 (60), REWIND (61), AMPLITUDE-O (62), and SOUL (5)) demonstrated MACE reductions of 12%–27% over 3 to 5 years; SELECT in obesity without diabetes (6) demonstrated 20% MACE reduction over a median of 39.8 months. Curves typically separate after year 1 and continue diverging through at least year 3; whether benefit widens beyond 5 years is unknown. FLOW (7) demonstrated 24% reduction in composite kidney outcome and 29% reduction in CV death over a median of 3.4 years in T2D with CKD, with the kidney curve separating from placebo within year 1. The SELECT renal substudy (86) extended renal benefit to non-diabetic obesity with CVD; the FLOW SGLT2i subgroup analysis (87) indicates that renal benefit is preserved on baseline SGLT2i therapy. Benefit accrues in proportion to time on therapy; the trajectory observed in trials depends on continued exposure.

### 5.3 Hepatic, OSA, and HFpEF trajectory

SYNERGY-NASH, a phase 2 trial (8), demonstrated tirzepatide’s effect on MASH histological resolution and fibrosis at 52 weeks. The ESSENCE phase 3 program for semaglutide has now reported 72-week interim resolution and fibrosis improvement data (141) on the basis of which the FDA granted accelerated approval in August 2025 for non-cirrhotic MASH F2 to F3 (142). The earlier phase 2 semaglutide data (79) establish a directionally consistent signal for MASH resolution. The trajectory beyond 1 year is not yet defined for either agent, and the semaglutide cirrhosis trial (80) established that benefit is not observed in compensated cirrhosis. Furthermore, population-level cohort evidence supports reduced cirrhosis progression in MASLD without established cirrhosis (102). Obstructive sleep apnea with obesity has shorter-horizon data: SURMOUNT-OSA (4) demonstrated AHI reduction at 52 weeks with cardiometabolic improvement attributable in part to OSA-specific mechanisms beyond weight loss (77); durability over chronic OSA management is unresolved. For HFpEF, the STEP-HFpEF program (72–74) and SUMMIT (75) established symptom, functional, and clinical-event improvement with weight loss and circulatory volume reduction; the SUMMIT secondary analysis (3) demonstrated reductions in systolic blood pressure, estimated blood volume, C-reactive protein, and troponin T. The trajectory question across all three indications is whether benefit is sustained beyond 1 to 2 years.

### 5.4 Durability of response and the plateau question

Why does weight loss plateau? The appetite-suppressing effect is balanced by physiological adaptations defending body weight—reduced resting energy expenditure, increased hunger signaling, adipose-tissue hormone secretion—producing a new equilibrium at a lower weight, defended for as long as the agent continues. The on-treatment phase is a steady state, not continuing decline. Clinicians and patients expecting continued loss beyond month 18 are often disappointed, but the disappointment is not a sign of failure. What to do at the plateau (increase dose, switch agents, or hold therapy in place) is among the most clinically consequential questions trial literature has not addressed.

### 5.5 Cardiovascular benefit mechanism question

The cardiovascular and renal trajectory discussion above established what happens to MACE on therapy, with 12%–27% reduction across the class accruing over years. How that reduction is produced has been answered two ways. A substantial fraction of the cardiovascular benefit is weight independent (drawing on SELECT and SUMMIT mediation and secondary analyses, though the mediation reasoning is observational and *post-hoc* and cannot prove a mechanism independent of weight), and the available evidence supports this position over the alternative that benefit is largely downstream of weight reduction.

Three lines of evidence converge. First, early CVOTs showed a mismatch between modest weight loss and MACE reduction: LEADER, SUSTAIN-6, and REWIND produced MACE reductions of approximately 12%–26% despite only modest weight differences of roughly 1.5–5 kg (59–61). Second, mediation analyses support only partial mediation by conventional risk factor or adiposity changes. In SELECT, approximately 33% of the observed MACE benefit was estimated to be mediated through waist circumference reduction, while the cardioprotective effect was independent of baseline adiposity and body-weight loss. Third, tissue distribution studies identify a plausible mechanism. Liraglutide reduces visceral and ectopic fat disproportionately relative to total body weight loss (41), and tirzepatide reduces inflammatory biomarkers such as C-reactive protein and interleukin-6 (39), though the inflammatory biomarker reductions co-occur with weight loss and should not be characterized as fully weight-independent.

SELECT provides the strongest available evidence for a substantial weight-independent CV benefit in the non-diabetic obesity population, though mediation and secondary analyses are observational and *post-hoc* and cannot prove mechanism independent of weight. Mean weight loss approximately 9% sustained over 4 years, MACE reduction 20% (6, 100). The heart-failure subgroup analysis (76) extended this benefit across both preserved and reduced ejection fraction phenotypes. The cardiovascular effect cannot be accounted for by weight loss of that magnitude alone.

The clinical implication is that cardiovascular outcome data should drive prescribing in patients with cardiovascular risk even when expected weight loss is modest. Pending evidence that will adjudicate the relative contribution of mechanism and weight in the highest-efficacy agent is the completion of tirzepatide’s dedicated cardiovascular evidence base [SURPASS-CVOT and SURMOUNT-MMO (22)].

## 6 Body composition, nutrition, and the metabolic–functional question

### 6.1 Body composition findings across the class

Body composition data now characterize the typical pattern of loss. In the SURMOUNT-1 DXA substudy, ~75% of weight lost on tirzepatide was fat mass and ~25% lean mass, with the proportion stable across sex, age, and weight-loss tertile (103). The SURPASS-3 MRI substudy in T2D reached a similar conclusion: muscle volume reductions were consistent with what age and weight loss would predict, while muscle fat infiltration reduced more than predicted in the metabolic-quality direction (104). The pooled GLP-1 receptor agonist literature converges on the same pattern: lean mass loss is observed, proportional to total weight loss, and qualitatively similar to what is observed after equivalent weight loss with caloric restriction alone (105). The framing that incretin therapy causes sarcopenic obesity has accordingly been overstated. In the average trial-population adult, the DXA and MRI pattern is consistent with adaptive remodeling. The SEMALEAN cohort reported handgrip strength improvement (+4.5 kg at 12 months) and a reduction in sarcopenic-obesity prevalence from 49% to 33% under semaglutide of 2.4 mg (147). Older adults, adults with pre-existing sarcopenia, and adults with modest baseline muscle reserve may not follow this pattern.

### 6.2 Functional-endpoint gap

Current reassurance about lean mass rests on DXA mass proportionality and MRI muscle composition analysis. The long-term functional implications of lean mass changes under sustained incretin therapy remain an area of active investigation. Functional outcomes that would detect sarcopenic harm in vulnerable subgroups (gait speed, grip strength, chair-rise time, and time-up-and-go) are largely absent from the pivotal trial literature (104–106). The 6-min walk distance has been measured in the STEP-HFpEF program (73, 74), but those are disease-specific populations where the outcome is part of the indication; the gap is for primary-prevention populations who are the largest treated group. This is not a reason to adopt the sarcopenic-obesity framing prematurely. It is a reason to specify functional endpoints that future trials should capture, particularly in adults at structural risk for functional decline.

### 6.3 Subpopulations warranting specific prescription

Five populations make the metabolic-functional question most clinically consequential. Adults aged 65 years and older are the largest; the trial literature above 75 years is limited. Sarcopenia at initiation, defined by lean mass below age- and sex-adjusted thresholds, is second. Frailty risk identified by the Fried phenotype or equivalent is a third. Planned future surgery is fourth: lean-mass reserve matters for postoperative recovery. The fifth is concurrent catabolic illness (advanced cancer, advanced chronic kidney disease, and advanced heart failure), where the underlying disease is itself driving lean-mass loss that the agent will compound.

The clinical implication was stratified prescription. Protein supplementation of approximately 1.0 to 1.2 g per kilogram of adjusted body weight per day in adults with obesity during active weight loss, structured resistance exercise two to three times per week, and monitoring of functional status are reasonable additions to therapy in these populations. None has been formally tested in a trial that included incretin receptor agonist therapy, but the general principles from the sarcopenic obesity literature (106) support the framework.

### 6.4 Myostatin and activin pathway combination program

The pharmacological response has been investigational agents targeting myostatin and activin pathways, combined with incretin therapy in preclinical and early-phase studies to test whether lean mass can be preserved or enhanced during weight loss (107, 108). These combinations remain investigational, and their functional value on clinically meaningful endpoints such as strength, gait speed, and fall risk is unproven. Bimagrumab is among the most advanced agents in this space, with phase 2 combination data showing that bimagrumab plus semaglutide can augment fat-mass reduction while preserving lean mass. The pipeline is competitive, and the functional value of lean-mass preservation remains uncertain. These agents may be most useful for stratified subpopulations in whom lean-mass preservation has clear functional or metabolic relevance, but their incremental benefit for the average patient who loses weight without functional decline is unclear. Combination therapy is therefore a stratification opportunity rather than a categorical adjunct.

### 6.5 Metabolic–functional question reframed

Complementary real-world evidence in tirzepatide-treated adults with overweight or obesity indicates that adherence to a Mediterranean dietary pattern, assessed by the validated PREDIMED score, is associated with greater reduction in visceral adiposity index and improved insulin sensitivity independent of weight change over 3 months and that adiposity indices beyond BMI (waist-to-height ratio, a body shape index, body roundness index, and visceral adiposity index) capture metabolic-quality shifts that BMI alone does not (149). Visceral adipose reductions with liraglutide (41) and muscle-quality improvements in SURPASS-3 MRI (104) suggest the metabolic-functional question is most usefully reframed as a body-fat-distribution question. The cardiometabolic benefit comes from a metabolic-quality shift, not a total-mass reduction alone, and that reframing helps explain the dose–effect mismatch discussed earlier under the cardiovascular benefit mechanism question. The synthesis position: lean-mass loss is largely adaptive and proportionate to total weight loss in the average adult. The specific subpopulations identified above warrant protein and resistance-exercise prescription, with myostatin pathway combination therapy where lean-mass risk is structural. The priority is thus to specify functional endpoints in future trials, particularly in older adults and frailty-risk patients.

## 7 Adverse-event landscape and emerging signals

### 7.1 Gastrointestinal events

The gastrointestinal profile is the most commonly reported safety concern and is treated above in the titration and tolerability discussion. Briefly, ≥1 gastrointestinal adverse event was reported in approximately 28%–73% of tirzepatide-treated participants across SURMOUNT-1 to -4 (95). The events were mostly mild to moderate, predominantly during dose escalation, and often attenuated over time, consistent with clinical adaptation and tachyphylaxis of the gastric emptying effect (36, 37, 50). Treatment discontinuation due to GI adverse events ranged from approximately 1% to 8% across SURMOUNT tirzepatide arms (95). Semaglutide also produces a substantial gastrointestinal adverse event burden in obesity trials STEP-1 and STEP-5 (68, 99).

### 7.2 Hepatobiliary events

Gallbladder and biliary disease occur at higher rates with GLP-1 receptor agonists than with placebo or active comparators. The He 2022 systematic review estimated a relative risk of 1.37 for the composite gallbladder and biliary disease endpoint, with higher risk at higher doses, longer duration, and the weight-loss indication versus T2D (26). The mechanism combines biliary stasis from delayed gallbladder emptying and the cholelithiasis that accompanies rapid weight loss from any cause (37). The clinical implication is dose- and indication-dependent counseling and a lower diagnostic threshold for right upper quadrant or epigastric symptoms in the first year of therapy.

### 7.3 Pancreatic events

The pancreatitis signal from early exenatide development has not been confirmed in subsequent adjudicated trial data. Adjudicated randomized trial data show no significant excess of acute pancreatitis (OR 0.745, 95% CI 0.47 to 1.17) (25, 109). Some large real-world observational sources show a modest signal that may reflect residual confounding or diagnostic bias (29). Pancreatic cancer signal has not been replicated; real-world data may be biased toward reduced incidence in obese/diabetic populations using these agents (110). History illustrates the broader pattern: regulatory flag, epidemiological adjudication, and partial or complete defusion.

### 7.4 Thyroid signals

The rodent thyroid C-cell tumor signal identified during preclinical development of liraglutide (24) remains the basis for the US boxed warning. Two decades of human experience have not replicated it at the population level. The Morales 2025 multi-database cohort study reported that GLP-1 receptor agonist exposure was not associated with an increased risk of thyroid tumors compared with SGLT2is, DPP-4is, or SUs (111). The cancer meta-analysis (48 trials) of Ko (2026) reached the same conclusion at moderate certainty (110). The boxed warning should be read as a residual species-translation concern; personal or family history of medullary thyroid carcinoma or multiple endocrine neoplasia type 2 remains a formal labeled contraindication based primarily on rodent toxicology and unresolved species translation rather than proven human population risk (112).

### 7.5 Nonarteritic anterior ischemic optic neuropathy signal

The first major emerging signal of this decade is the association of semaglutide with NAION as reported by Hathaway and colleagues in a single-center matched cohort, with hazard ratios of 4.28 in type 2 diabetes and 7.64 in obesity (27). Subsequent replication has produced a heterogeneous picture. A Military Health System cohort of over 1.2 million beneficiaries reported that among adults with type 2 diabetes prescribed with semaglutide the risk of NAION was lower than in adults prescribed with non-GLP-1 comparators (odds ratio 0.36, 95% CI 0.25 to 0.51) (140). Other studies produced smaller but directionally consistent estimates: the OHDSI network analysis found a more modest increase in type 2 diabetes (113), the Danish–Norwegian active-comparator cohort reported HR 2.81 versus SGLT2 inhibitors (114), and a US target-trial emulation identified elevated risk for semaglutide or tirzepatide versus other antidiabetic agents (115). Absolute risk is low in all studies. The clinical implication is to mention the association at initial counseling, treat acute monocular vision loss as an indication to discontinue and refer urgently, and recognize that the signal is real but small enough such that it should not deflect prescribing in patients for whom the cardiovascular and metabolic indication is strong.

### 7.6 Neuropsychiatric signal: Pharmacovigilance versus evidence

The EMA raised a signal of suicidal ideation and self-injury during GLP-1 receptor agonist therapy in 2023. Subsequent epidemiological evidence has not supported it. The Hurtado 2024 propensity-weighted cohort against an SGLT2 inhibitor comparator found no elevated risk of suicidal ideation or self-injury (28). The VA-based atlas of 175 outcomes by Xie (2025) documented reduced rather than increased rates in several neuropsychiatric categories (29). The contemporary safety synthesis by Jalleh et al. reaches the same conclusion (30).

Current epidemiological evidence does not support a population-level increase in suicidal ideation or self-injury, but continued pharmacovigilance remains appropriate. The labeled contraindication for personal or family history of medullary thyroid carcinoma or multiple endocrine neoplasia syndrome type 2 remains formally in effect. Routine clinical screening for depression and suicidal ideation remains appropriate, both because the prevalence in the indicated population is high and because pharmacovigilance may yet identify specific subpopulations in whom a true association exists.

The signal’s persistence in clinical and public conversation after epidemiological adjudication largely defused it is itself a feature of this class’s safety landscape. Early signals raised during development have repeatedly outlasted the evidence that addressed them. Thyroid C-cell tumors, the 2010s pancreatitis signal, and the 2023 suicidality signal are canonical. **Table 2** presents the adverse-event landscape of selective GLP-1 receptor agonists and tirzepatide.

**Table 2:** Adverse-event landscape: selective GLP-1 RAs vs. tirzepatide.

Adverse event category	Selective GLP-1 RAs (class)	Tirzepatide (dual GIP/GLP-1 RA)	Principal source	Clinical implication
Gastrointestinal events (nausea/vomiting/diarrhea/constipation)	Nausea 15–44% (T2D doses vs. obesity 2.4 mg), vomiting 8–24%, diarrhea 10–32%; dose- and titration-dependent	27%–73% of participants reporting any GI event across SURMOUNT 1–4; modestly higher than semaglutide at max doses	SURPASS/STEP/SURMOUNT pooled (95)	Cluster during 16–20 weeks of titration; gastric-emptying tachyphylaxis develops; 4%–10% discontinue for intolerance (11, 12, 95)
Hepatobiliary (gallbladder/biliary disease)	RR 1.37 (95% CI 1.23–1.52) for composite gallbladder/biliary endpoint; dose- and indication-dependent (RR 2.29 in obesity trials vs. RR 1.27 in T2D trials)	Consistent with class; specific estimate limited	He 2022 SR/MA of RCTs (26); Jalleh 2024 motility review (37)	Lower diagnostic threshold for right-upper-quadrant or epigastric symptoms in the first year of therapy
Pancreatic (acute pancreatitis/pancreatic cancer)	Adjudicated trial data show no excess of acute pancreatitis (Peto OR 0.745, 95% CI 0.47–1.17); real-world atlas shows modest excess; pancreatic cancer signal not replicated Ko et al. found moderate-certainty evidence that GLP-1 receptor agonists probably have little or no effect on pancreatic cancer risk, with OR 0.84; 95% CI 0.53–1.35 (110).	No tirzepatide-specific pancreatic-cancer signal has been established in trials to date	Adjudicated RCT data: Storgaard 2017 (25), Monami 2017 (109); real-world: Xie 2025 atlas (29); cancer SR/MA: Ko 2026 (110)	Prior pancreatitis is not a formal class contraindication but warrants individualized risk–benefit discussion; counsel on persistent severe abdominal pain and discontinue/evaluate if pancreatitis is suspected
Thyroid (C-cell/medullary/differentiated)	No human population-level signal for medullary or differentiated thyroid cancer over two decades of exposure; thyroid cancer SR/MA OR 1.37 (95% CI 0.82–2.31), confidence interval crosses 1.0	No signal	Bjerre Knudsen 2010 rodent C-cell (24); Morales 2025 multi-database cohort (111); Ko 2026 SR/MA (110)	Boxed warning reads as residual species-translation concern; Personal/family history of MTC or MEN2 remains a formal labelled contraindication, based on rodent toxicology and uncertain human relevance (112)
Nonarteritic anterior ischemic optic neuropathy (NAION)	Semaglutide has a reproducible but rare observational NAION signal. Estimates vary by design: Danish T2D 5-year cohort HR 2.19; Danish-Norwegian active-comparator cohort HR 2.81; Hathaway single-center cohort HR 4.28 in T2D and 7.64 in obesity; OHDSI analysis found a more modest signal, including SCCS IRR 1.32, while one separate TriNetX/AJO study reported no increased risk, including RR 0.70 in T2D. Absolute risk remains low	Wang 2025 reported semaglutide or tirzepatide combined was associated with NAION versus other antidiabetic agents, HR 1.76; 95% CI 1.01–3.07, with absolute risk 0.04% vs. 0.02% over 2 years. A stable tirzepatide-specific NAION estimate was not separately established	Hathaway 2024 (27); Cai 2025 (113); Simonsen 2025 (114); Wang 2025 (115)	Counsel about the rare association at initiation. Acute monocular vision loss should prompt holding/discontinuing therapy pending urgent ophthalmology or neuro-ophthalmology evaluation
Neuropsychiatric (suicidal ideation/self-injury/depression)	No elevated risk vs. SGLT2 inhibitor comparator; several categories (substance use, psychotic, neurocognitive disorders) reduced rather than increased	No signal	Hurtado 2024 propensity-weighted cohort (28); Xie 2025 atlas (29); Jalleh 2026 safety synthesis (30)	Continued pharmacovigilance and routine clinical depression screening remain appropriate; current evidence does not support categorical attribution
Periprocedural (retained gastric content/aspiration)	Elkin 2025 found no significant association with pulmonary aspiration, OR 1.04, 95% CI 0.87–1.25, but increased residual gastric contents, OR 5.96, 95% CI 3.96–8.98 Approximately sixfold higher odds of residual gastric contents on imaging/endoscopy, without a demonstrated increase in pulmonary aspiration in pooled observational data	Same pattern; titration-period most pronounced; tachyphylaxis attenuates effect after 12 weeks	Elkin 2025 SR/MA (123); Jalleh 2024 review (50); ASA consensus (122)	Individualized management by symptoms, escalation phase, dose, procedure/anesthesia risk, and POCUS availability; avoid blanket withholding for all stable users

## 8 Persistence, discontinuation, and lifecycle interruptions

### 8.1 Real-world persistence and discontinuation

Real-world persistence is substantially lower than in pivotal trials: The largest US commercial-insurance cohort (obesity, no diabetes) found 32% persistence at 12 months and 47% at 6 months, with semaglutide being highest at 1 year (47%) and liraglutide being lowest (19%) (11). The large EHR cohort with both diabetes and non-diabetes populations is similar but stratified: 1-year discontinuation reached 65% in obesity without diabetes and 47% in obesity with type 2 diabetes (12). Danish nationwide registry data identified 21% discontinuation at 12 months among diabetes patients between 2007 and 2020, with adherence holding at 49% (116). UK primary care reported 41% 1-year discontinuation, with subcutaneous semaglutide and lower-titration phenotype faring worse than dulaglutide (19).

In the United States, the reasons for stopping are dominated by cost rather than therapeutic failure. Outside the United States, where list price is not the dominant patient-facing determinant, cost operates instead through payer formulary rules and reimbursement restrictions, and non-US persistence data [Lassen 2024 Danish nationwide cohort (116)] identify lower household income, lower educational attainment, and comorbidity burden rather than out-of-pocket price as independent discontinuation predictors. The Gasoyan EHR cohort reported cost or insurance coverage as the single most common reason (47.6%), side effects as he second (14.6%), and supply disruption as the third (11.8%) (18). These three together account for approximately three-quarters of discontinuation events (18, 117).

Persistence varies by demographic and socioeconomic factors trial literature does not capture: The Danish cohort identified lower household income, lower educational attainment, and Charlson comorbidity index above 3 as independent predictors of discontinuation (116). Higher household income (>$80,000/year) was protective in the US EHR cohort, particularly in T2D (12). The pattern is consistent: patients who continue therapy are systematically those who can afford to continue, not necessarily those in whom indication and benefit are highest.

### 8.2 Withdrawal and regain dynamics

The regain trajectory after abrupt discontinuation is well characterized: SURMOUNT-4, the most informative single source, enrolled 670 adults who had achieved a mean 20.9% weight reduction during a 36-week open-label tirzepatide lead-in. After randomization to continued tirzepatide or placebo, the tirzepatide group lost an additional 5.5% over 52 weeks, while the placebo group regained 14% (16). In terms of maintenance of ≥80% of lead-in loss, 89.5% continued tirzepatide vs. 16.6% placebo. STEP-4 demonstrated the analogous pattern for semaglutide: placebo-switched participants regained 6.9% over 48 weeks compared with continued 7.9% additional loss (118). SCALE Maintenance established the principle in the liraglutide era (119).

The STEP-1 trial extension followed the participants for 1 year after coordinated discontinuation and demonstrated regain of approximately two-thirds of weight loss, with reversion of systolic blood pressure, lipid panel, HbA1c, and waist circumference toward baseline (14) (**Figure 4**). The Berg 2025 meta-analysis confirmed that regain is proportional to original loss: 2.20 kg with liraglutide and 9.69 kg with semaglutide/tirzepatide (15). The regain trajectory is non-linear; the first 6 months account for most regain.

**Figure 4:**
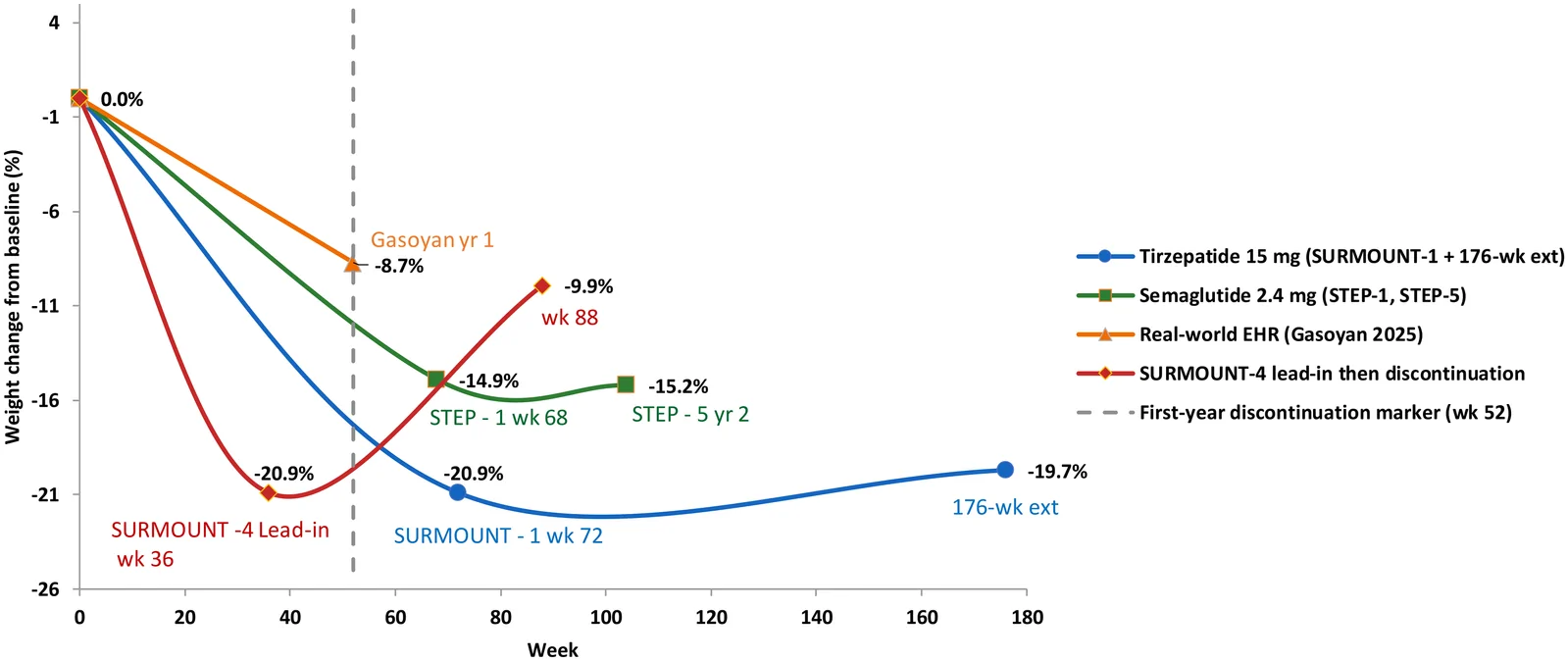
Weight-loss trajectory: trial efficacy versus real-world effectiveness, published primary-trial endpoints. Mean percentage change in body weight from baseline at every published primary-trial endpoint across four trajectories. Blue circles, tirzepatide 15 mg: SURMOUNT-1 week 72 (−20.9%, efficacy estimand) and 176-week extension (−19.7%). Green squares, semaglutide 2.4 mg: STEP-1 week 68 (−14.9%) and STEP-5 week 104 (−15.2%). Orange triangles, real-world EHR cohort: 1-year weight change (−8.7%) in 7,881 adults treated with semaglutide or tirzepatide for obesity (13). Red diamonds, SURMOUNT-4 lead-in then discontinuation: end of 36-week tirzepatide lead-in (−20.9%) and placebo-arm net change at week 88, 52 weeks after randomization to placebo (−9.9%). Lines connect within-series anchor points; no values are interpolated. The vertical dashed marker at week 52 indicates the first-year discontinuation window, the modal real-world observation point. The dotted callout highlights the rapid reversion of benefit following discontinuation. The trial-versus-real-world gap at week 52 and the post-discontinuation regain together illustrate that trial-magnitude weight reduction is preserved only under continued exposure and that real-world adherence is the principal determinant of effectiveness at the population level.

Available regain evidence is almost entirely on abrupt discontinuation: Planned tapering, dose reduction to maintenance, intermittent dosing, and agent switching are mechanistically different from abrupt cessation and have not been formally characterized. Whether cardiometabolic benefit reverts at the same rate as weight reverts is also not well characterized. Current evidence supports chronic-pharmacotherapy framing only under continuous therapy at maintenance dose.

### 8.3 Reinitiation and the cyclical-use pattern

Discontinuation is rarely permanent: Rodriguez et al. followed 41,792 EHR discontinuers and quantified reinitiation: 47.3% with T2D and 36.3% without T2D had restarted within 1 year (12). Weight regain was the strongest reinitiation predictor (each 1% regain: 2.3%–2.8% higher hazard of restart). Cyclical use has become a common pattern among adults who discontinue and then restart, and the trial-defined model of continuous therapy from initiation to indefinite maintenance describes a minority of treated patients.

Whether cyclical use yields comparable cardiometabolic benefit is unknown. The clinical questions are practical, namely: whether benefit accumulates with each on-treatment period or attenuates with repeated cycling, whether tachyphylaxis develops with repeated initiation, and whether agent switching during a reinitiation cycle preserves the response. None has been formally tested. The review’s position is that cyclical use deserves a dedicated trial design, which is developed in the research agenda below.

### 8.4 Cardiovascular signal after discontinuation, with reverse-causation caveat

Observational data link GLP-1 RA discontinuation to elevated MACE. The Piccini cohort of 550 patients with type 2 diabetes over a median of 5 years reported discontinuation independently associated with elevated MACE risk in both primary and secondary prevention, with hazard ratios of 3.40 and 2.71, respectively (17). The Danish target-trial emulation reached a directionally consistent conclusion: sustained GLP-1 RA exposure was associated with 2.5% absolute reduction in 3P-MACE over 3.5 years versus sustained DPP-4 inhibitor exposure (120). The interpretive caveat is substantial: discontinuers may differ from continuers in ways that themselves predict cardiovascular events; reverse causation cannot be ruled out. The signal is consistent with, but does not establish, a causal relationship.

This caveat strengthens rather than weakens the case for randomized continuation-versus-withdrawal trials. The fundamental question is whether the cardiometabolic benefits accumulated during 1 or 2 years of therapy are forfeited at discontinuation. In the absence of confounding by health deterioration, this question cannot be answered by available observational designs. Industry-sponsored withdrawal RCTs exist at the weight and cardiometabolic-marker level (SURMOUNT-4 and STEP-4), but multi-year post-discontinuation CV and renal outcome trials do not, reflecting both cost and asymmetric commercial incentive of designs characterizing what is lost on cessation. Closing this gap is plausibly a non-industry funding priority.

### 8.5 Chronic-pharmacotherapy framing, its access dimension, and the research gap

The cumulative picture supports a specific framing. These are chronic pharmacotherapy. Benefits accrue over years of continued exposure, weight and cardiometabolic parameters revert toward baseline within months of abrupt cessation, the cyclical pattern in routine care does not approximate trial-defined continuous therapy, and the reasons patients discontinue are not primarily clinical but economic, intolerance-driven, and supply-driven.

This framing has consequences. If long-term cardiometabolic benefit requires continuous exposure at therapeutic dose at a current US out-of-pocket cost of approximately $1,000 to $1,500 per month, the access gradient documented in registry data and the cascade-of-care disruptions during the 2022 to 2024 semaglutide shortage become structural rather than incidental features of the therapeutic landscape (20, 121). Compounded semaglutide and tirzepatide products emerged outside the regulatory pathway during the shortage and cost barrier, with FDA action in 2024 narrowing but not closing the supply channel. NHS access pathways and EU member-state formularies have adopted more restrictive prescribing criteria than US private insurers, which produces a different access gradient but does not solve the underlying problem. The critique is real; resolution requires policy decisions beyond this review’s analytical scope.

The most important research gap is the absence of trials testing what happens when therapy is changed rather than maintained. The five specific trial designs are developed in the research agenda that follows: a continuation-versus-withdrawal RCT in sustained responders at year 2; a full-dose-versus-reduced-dose maintenance RCT in similar responders; structured drug-holiday and restart protocols with defined cycling schedules; agent-switching protocols between selective GLP-1 receptor agonists, dual incretin agonists, and oral non-peptide GLP-1 receptor agonists; and the dedicated tirzepatide cardiovascular outcome trial (22). None of the first four exists. **Table 3** presents the real-world persistence, titration, and outcome studies for GLP-1 receptor agonists.

**Table 3:** Real-world persistence, titration, and outcome studies for GLP-1 receptor agonists.

Study	Database/population	Sample size	12-month persistence (or 1-year discontinuation)	Key finding/reasons
Gleason 2024 (11)	US commercial insurance (Prime Therapeutics), obesity without diabetes, new initiators 2021	4,066	32.3% overall persistence at 12 months; 46.3% at 180 days; 1-year persistence highest with semaglutide 47.1% and lowest with liraglutide 19.2%	Mean PDC 51.0% during 1-year assessment; 27.2% adherent (PDC ≥ 80%); 11.1% switched GLP-1 agents within first year; poor 1-year persistence in adults without diabetes
Rodriguez 2025 (12)	US EHR network (Truveta), adults with overweight or obesity, new initiators 2018–2023	125,474	1-year discontinuation: 64.8% without T2D and 46.5% with T2D; equivalent persistence 35.2% and 53.5%	Among 41,792 discontinued patients with discontinuation-weight data, 1-year reinitiation was 36.3% without T2D and 47.3% with T2D. Greater weight loss was associated with lower discontinuation; moderate/severe GI AEs were associated with higher discontinuation; weight regain was associated with reinitiation; income >$80,000 was protective against discontinuation in T2D.
Gasoyan 2025 (18)	US EHR (Cleveland Clinic), obesity, semaglutide or tirzepatide initiators; manual chart review of 288 randomly selected discontinuers	288 (chart-review sample; 145 semaglutide, 143 tirzepatide)	Not a primary persistence outcome	Reasons for stopping (*n* = 288): cost or insurance coverage 47.6% (*n* = 137); gastrointestinal intolerance 14.6% (*n* = 42); supply disruption 11.8% (*n* = 34); switched to compounded medication 2.4% (*n* = 7); unsatisfactory weight loss 1.7% (*n* = 5); other reasons 10.8% (*n* = 31); reason unspecified in EHR 11.1% (*n* = 32). Cost the most common reason at every time point
Lassen 2024 (116)	Danish nationwide registries, type 2 diabetes, first-time GLP-1 RA users 2007–2020	44,343	78.8% persistence at 12 months (21.2% discontinuation); 14.2% discontinuation by 6 months; adherence (MPR > 0.8) 50.4% at 6 months and 48.6% at 12 months	Independent predictors of discontinuation: lower household income, lower educational attainment, Charlson comorbidity index >3
Ulrich 2026 (19)	UK primary care, IQVIA THIN/IMRD; adults with T2D initiating GLP-1RAs, 2018–2023	8,200	41% 1-year discontinuation overall; 42% in established CVD subgroup	Only 58% of subcutaneous or oral semaglutide initiators reached recommended maintenance dose by second prescription (4 weeks); 21% remained on starting dose; substantial real-world titration shortfall
Rodriguez 2024 (98)	US EHR network, adults with overweight or obesity, semaglutide vs. tirzepatide propensity-matched	18,386 matched (9,193 pairs) from 41,222 total cohort	Follow-up ended by discontinuation in 55.9% receiving tirzepatide and 52.5% receiving semaglutide	At 12 months on-treatment: tirzepatide −11.4% (95% CI −12.0 to −10.8) vs. semaglutide −6.2% (95% CI −6.7 to −5.8); adjusted difference −6.9% (95% CI −7.9 to −5.8). Tirzepatide more likely to achieve ≥5% (HR 1.76, 95% CI 1.68–1.84), ≥10% (HR 2.54, 95% CI 2.37–2.73), and ≥15% (HR 3.24, 95% CI 2.91–3.61) weight loss; GI AE rates were similar between groups
Samuels 2025 (96)	Single academic multidisciplinary obesity clinic, retrospective cohort 2022–2024	2,306	Median persistence 10.7 months (IQR 5.4–16.3); 81% of semaglutide users escalated to ≥1 mg, 23% to 2.4 mg; 75% of tirzepatide users reached ≥10 mg, 28% reached 15 mg	Median weight loss 9.4% (IQR 6.0–13.4) at ≥6-month persistence; 14.4% (IQR 9.5–20.5) at ≥12-month persistence; structured titration in obesity-clinic setting approximates phase 3 weight-loss figures
Piccini 2023 (17)	Italian single-center, type 2 diabetes ± prior CV disease	550 (395 primary prevention; 155 secondary prevention)	Not a primary outcome; median follow-up 5.0 years (primary prevention) and 3.6 years (secondary prevention); median treatment 3.2 years (primary prevention) and 2.5 years (secondary prevention)	Time-dependent GLP-1 RA cessation associated with HR 3.40 (95% CI 1.82–6.32) for MACE in primary prevention and HR 2.71 (95% CI 1.46–5.01) in secondary prevention; observational with reverse-causation interpretation
Sørensen 2025 (120)	Danish nationwide target-trial emulation, type 2 diabetes meeting LEADER criteria	25,753 (6,681 GLP-1 RA + 19,072 DPP-4i)—figure cited; broader paper-level cohort 50,815 (16,619 + 34,196) is the unrestricted analysis	Not a primary outcome; sustained 3.5-year exposure modelled	Sustained GLP-1 RA exposure associated with 2.5% (95% CI 0.8%4.1%) absolute reduction in 3P-MACE vs. sustained DPP-4 inhibitor exposure over 3.5 years; additional absolute reductions for CV mortality 2.3% (1.4–3.1), all-cause mortality 2.5% (0.7–4.3), heart failure 0.9% (0.01–1.8); supports value of continuous therapy

## 9 Practical lifecycle problems

### 9.1 Periprocedural management

The gastric-emptying effect has raised a periprocedural question that has been the subject of regulatory and clinical attention since 2023. The ASA 2023 consensus guidance recommended withholding daily-dose agents on the procedure day and weekly-dose agents for 1 week before elective procedures requiring general anesthesia (122). Subsequent evidence has refined this. The position taken here is that periprocedural management should be individualized by setting and titration status rather than handled with a blanket withholding policy.

Pooled observational evidence produces two consistent findings. The systematic review of 28 studies including 466,373 patients (471 aspiration cases) by Elkin (2025) found no association with peri-operative pulmonary aspiration (pooled odds ratio 1.04, 95% CI 0.87 to 1.25) (123), but a substantial association with residual gastric contents on imaging (pooled odds ratio 5.96, 95% CI 3.96 to 8.98), with withholding at least one dose halving the residual-content risk (123). The meta-analysis of endoscopy-specific data by Facciorusso (2024) reached a similar conclusion: approximately fivefold higher residual gastric contents, with higher aborted-procedure rates but no detectable increase in clinical aspiration (124). The clinical interpretation: the residual-content signal is real, the aspiration signal is not.

In setting-specific operationalization (123, 125, 126), upper endoscopy under sedation carries moderate aspiration risk, gastric point-of-care ultrasound is appropriate when symptomatic or recently initiated (<12 weeks), and standard fasting with a clear-liquid pre-procedure diet usually suffices for long-stable users. Non-gastrointestinal surgery under general anesthesia is of a higher risk in recently initiated or symptomatic patients (POCUS or 1-week withholding reasonable; standard fasting often sufficient for long-stable users). Elective procedures generally carry the lowest risk; clear-liquid protocols on the day before usually suffice for stable users on therapy for more than 12 weeks.

The titration-versus-maintenance distinction matters. Retained gastric contents are most pronounced during the first 12 weeks of titration and attenuate substantially as tachyphylaxis develops (36, 50). The original ASA guidance treated long-stable patients as equivalent to recent initiators, which is the opposite of what the pharmacology predicts. Periprocedural risk is highest during the titration window when tachyphylaxis has not yet developed, and both the tolerability profile and the residual-gastric-content signal attenuate together as tachyphylaxis develops over the first 12 weeks of therapy (36, 50).

Gastric POCUS is not universally available. The tiered approach proposed here reserves POCUS for higher-risk procedures (general anaesthesia, recent initiation, and symptomatic) and patients, with clear-liquid protocols sufficing for stable users undergoing lower-risk procedures. System-level POCUS training/access expansion is a structural priority (125). Multi-society guidance is moving toward this more permissive position (126).

### 9.2 Oral drug absorption and drug interactions

The gastric-emptying delay can modestly delay the peak plasma concentration of co-administered oral medications without affecting AUC during the titration window, and clinical practice has not produced a consistent signal of harm (36, 37). Caution and dose-timing strategies are reasonable for narrow-therapeutic-index drugs, oral contraceptives during dose escalation, and drugs with upper-GI absorption windows.

### 9.3 Special populations

Three groups warrant specific discussion: Older adults (≥65 years) tolerate therapy similarly to younger adults in trial populations, but the >75-year subgroup is small and >80-year data are sparse. The body-composition considerations discussed earlier apply with particular force in this group.

Pediatric and adolescent prescribing emerged over the past 5 years. SCALE Kids in children 6 to under 12 years (127), SCALE Teens in adolescents (97), and STEP TEENS in adolescents (23) tested efficacy over 56 to 68 weeks. Long-term safety data on bone accrual, pubertal development, and adult reproductive outcomes are not yet available in these age bands, although efficacy has been established in their respective age bands, with safety profiles broadly similar to adults. The lifecycle questions are sharper than in adults: the period of therapy required to convert reduced adolescent weight into reduced adult disease is potentially decades, and the maintenance-and-de-escalation evidence gap is correspondingly larger.

Pregnancy and lactation are scope-acknowledged gaps. Patients planning pregnancy should discontinue anti-obesity GLP-1/GIP therapy before conception. For planned pregnancy, the semaglutide label recommends discontinuation at least 2 months before conception. The tirzepatide label does not specify a formal preconception washout interval, but the approximately 5-day half-life supports discontinuation approximately 1 month before planned conception, informed by shared decision. On recognition of inadvertent pregnancy, the anti-obesity GLP-1 or GIP therapy should be stopped. Tirzepatide can reduce oral contraceptive exposure during initiation and dose escalation, so additional or non-oral contraceptive coverage should be provided during initiation and for 4 weeks after each dose increase, with pharmacokinetic guidance supporting an approximately 30-day washout for drug clearance. The bariatric–metabolic surgery postoperative setting is a related gap; whether and when to use incretin agents in patients with weight regain after surgery is an area of emerging clinical interest but has limited formal evidence.

## 10 Access, cost, and the system determinants of therapeutic durability

### 10.1 Cost-effectiveness, list price, and the out-of-pocket gradient

The pharmacoeconomic case is stronger than the access pattern suggests. The Tang et al. cost-effectiveness analysis of tirzepatide in type 2 diabetes with concomitant obesity used the BRAVO diabetes model over a 30-year time horizon. It estimated an incremental cost-effectiveness ratio of approximately $3,092 per QALY for the 10-mg dose and dominance for the 15-mg dose, with greater than 98% probability of cost-effectiveness at the $100,000 willingness-to-pay threshold (128).

The US list prices for injectable GLP-1/GIP drugs range from approximately $1,000 to $1,500 per month, though payer-negotiated and manufacturer self-pay prices reported in 2025 (148) and 2025–2026 are materially lower (e.g., a Medicare-wide $245 per month and starting-dose self-pay programs in the $199–$349 range), among the most expensive pharmacological therapies for chronic non-cancer conditions. Insurance coverage is the principal access determinant. In the T2D indication, coverage is broad; in obesity, it is variable and frequently restrictive; off-label or marginally on-label coverage is rare. The cost-effectiveness signal at the population level does not translate to access at the individual patient level, and the discontinuation-for-cost rate of 47.6% (18) reflects this mismatch rather than a deficiency of the pharmacoeconomic case.

### 10.2 Supply, shortages, and the compounded-products episode

The 2022 to 2024 semaglutide and tirzepatide shortage was the first major test of the class’ supply chain at scale. Demand outpaced manufacturing capacity, with the FDA listing both agents as in shortage during much of 2023 and early 2024. The shortage produced two lifecycle consequences. The first was acute discontinuation: supply disruption was the third most common reason for stopping (11.8%) (18), and the Ashruf cascade-of-care analysis documented increased prescribing disparities, with patients from lower-income areas and racial and ethnic minority groups disproportionately affected in the cancer-patient cascade-of-care cohort studied (20).

Second is the emergence of compounded products as a shadow-market response. Section 503A and 503B compounding pharmacies produced large quantities of compounded semaglutide and tirzepatide during the shortage, marketed through telehealth platforms at prices substantially below branded products. Clinical concerns included non-standardized dosing, differing formulation excipients and concentrations, and counterfeit product penetration in some jurisdictions. FDA action across late 2024 and the first half of 2025 formally ended large-scale shortage-era compounding. Tirzepatide shortage was declared resolved in December 2024 (with 503A and 503B enforcement discretion ending in February and March 2025, respectively), and semaglutide shortage was resolved in February 2025 (503A discretion ending in April and 503B in May). Smaller-scale and patient-specific compounding continued, and the regulatory framework remains under active litigation.

The episode revealed three structural features of the supply chain: Demand at population scale exceeds the manufacturing capacity the development pathway was designed to support. Price signals at the branded-product level are sufficiently far from cost signals at the underlying-molecule level that a shadow market is economically viable, with predictable quality consequences. The regulatory and prescribing framework was not designed for chronic pharmacotherapy at population scale, and the rapid expansion of telehealth-based prescribing during the shortage exposed the gap.

### 10.3 Disparities and the policy question

Access to incretin and multi-receptor agonist therapy is inequitably distributed. Lower household income, lower educational attainment, and higher comorbidity index predicted discontinuation in the Danish cohort (116); higher US household income was protective in the EHR cohort (12). The Ashruf analysis showed that prescribing disparities in the cancer-patient cascade-of-care cohort widened, not narrowed, during the supply disruption (20). NHS access pathways and EU formularies have adopted more restrictive on-label prescribing criteria than US private insurers, producing a different access gradient (rationing rather than affordability) but not solving the underlying problem of expensive chronic pharmacotherapy at population scale.

The policy questions are themselves clinical–scientific questions in a class whose benefit is contingent on continuous exposure: when access systematically excludes the populations in whom indication is highest, the trial-defined effect ceases to be the population-level effect, and treating access as external policy mis-locates where the limits of class efficacy lie. The relevant clinical and research observations are that access is a determinant of therapeutic durability, as developed earlier under persistence and discontinuation, the most expensive interventions are not necessarily those with the weakest cost-effectiveness case, and the system-level determinants of long-term benefits are at least as important as the pharmacological determinants for understanding what the class will accomplish at the population scale.

## 11 Lifecycle research agenda and outlook

### 11.1 Pipeline

Four pipeline categories will shape the next decade as discussed below.

Triple agonism (exemplified by retatrutide) extends multi-receptor activation to the glucagon receptor. The Jastreboff (2023) phase 2 trial in obesity demonstrated 24.2% weight loss at 48 weeks with the 12-mg dose, the highest pharmacological weight loss yet reported in a controlled trial (48). A phase 2a MASLD substudy demonstrated 81.4% to 82.4% relative liver fat reduction at 24 weeks with the 8- and 12-mg doses and complete liver fat normalization (<5%) in 89% and 93% of those participants, respectively, at 48 weeks (81). The TRIUMPH phase 3 development program is ongoing.

Oral non-peptide GLP-1R agonism (exemplified by orforglipron) removes oral semaglutide’s food-and-water-restriction requirement. ATTAIN-1 demonstrated 11.2% weight loss at 72 weeks with 36 mg in obesity (treatment-regimen estimand; 12.4% by the efficacy estimand) (129). ATTAIN-2 demonstrated 9.6% weight loss at 72 weeks with 36 mg in obesity with type 2 diabetes (130). ACHIEVE-1 demonstrated 1.48% HbA1c reduction and 7.9% weight loss at 40 weeks with 36 mg in early type 2 diabetes managed with diet and exercise alone (131). Higher-dose oral semaglutide (OASIS 1, 50 mg) has also reached the phase 3 efficacy range at 15.1% weight loss (132). Adherence improves with oral dosing in most chronic-disease classes, and supply-chain considerations differ from injectable peptides.

Amylin co-agonism, exemplified by CagriSema (cagrilintide–semaglutide), extends the multi-receptor framework beyond incretin biology. REDEFINE-1 demonstrated 20.4% weight loss at 68 weeks (treatment-policy estimand; 22.7% by the trial-product estimand) of a magnitude close to but not identical to tirzepatide (133). In the subsequent head-to-head REDEFINE-4 phase 3 trial, CagriSema achieved 23.0% weight loss at 84 weeks compared with 25.5% for tirzepatide 15 mg (efficacy estimand) but did not meet the pre-specified non-inferiority endpoint versus tirzepatide (Novo Nordisk company announcement, February 2026) (143).

Glucagon co-agonism (survodutide and pemvidutide) focuses on hepatic and energy expenditure mechanisms. The survodutide phase 2 trial in MASH with fibrosis stages F1–F3 (non-cirrhotic) demonstrated MASH resolution without worsening of fibrosis in 47%, 62%, and 43% of participants on 2.4, 4.8, and 6.0 mg, respectively, versus 14% on placebo (82, 134), with the dedicated MASH phase 3 program [LIVERAGE in non-cirrhotic MASH (135) and LIVERAGE-Cirrhosis in compensated MASH cirrhosis (146)] ongoing; the parallel obesity phase 3 program (SYNCHRONIZE-1) has reported 16.6% weight loss at 76 weeks. Pemvidutide reduced liver fat by up to 68.5% at 12 weeks in a phase 2 MASLD trial (83) and produced 15.6% weight loss at 48 weeks in the phase 2 MOMENTUM trial (136). Mazdutide phase 1b in Chinese adults supports broader geographic development (137).

A distinct direction is GIPR antagonism conjugated to GLP-1R agonism, exemplified by maridebart cafraglutide, where the phase 2 trial demonstrated up to approximately 20% weight loss at 52 weeks with once-monthly dosing and no weight plateau (139); its long-half-life design positions the agent to test whether benefit persists after dose discontinuation, a question now under formal evaluation in the trial’s ongoing extension and among the most direct clinical signals for the lifecycle-relevant potential of the bispecific GIPR antagonist approach (47). The Melson (2024) comprehensive pipeline review documents additional agents in earlier development (108).

### 11.2 Lifecycle research agenda

The most consequential research gap is not in the well-populated pipeline but in the lifecycle questions no current trial is designed to answer. Five trial designs are required, an four of them are not yet initiated.

The first trial design is a continuation-versus-withdrawal RCT in sustained year 2 responders. Patients with at least 10% weight loss maintained for 12 or more months would be randomized to continue full dose or to structured withdrawal, with cardiometabolic, hepatic, and weight outcomes at year 5. This design would resolve the reverse-causation question observational discontinuation literature cannot answer (raised in Section 8.4).

The second trial design is a full- versus reduced-dose maintenance trial at year 2. Once initial weight loss is stable, randomization to full versus reduced maintenance dose would test whether de-escalation is feasible without regain and whether a more affordable maintenance regimen could be designed.

The third trial design is a structured drug-holiday and restart protocol. A trial of structured intermittent dosing (for example, 6 months on followed by 3 or 6 months off, repeated) versus continuous dosing would test whether cyclical use produces the same cardiometabolic benefit and whether tachyphylaxis develops with repeated initiation.

The fourth trial design is an agent-switching protocol. Switching from semaglutide to tirzepatide is the dominant real-world pattern but has not been formally tested in patients who have failed on the first agent. A trial randomizing sub-optimal responders to continued first-agent therapy at maximum dose or switch to second-agent therapy would test whether within-class superiority observed in agent-naive populations applies to the population that has actually failed.

The fifth trial design is completion of the dedicated tirzepatide cardiovascular outcome program. SURPASS-CVOT in T2D and SURMOUNT-MMO in obesity without diabetes together comprise the cardiovascular evidence base for the highest-efficacy agent (22). SURPASS-CVOT, reported in 2025, demonstrated non-inferiority of tirzepatide versus dulaglutide for three-point MACE in type 2 diabetes with established atherosclerotic disease (12.2% vs. 13.1% over a median of 4 years; non-inferiority *P* = 0.003, superiority *P* = 0.09), with greater reductions in HbA1c, body weight, renal function decline, and all-cause mortality as key secondaries (138). The dedicated tirzepatide CVOT in the obesity-without-diabetes population (SURMOUNT-MMO) remains in follow-up, with results expected in 2027 (22). SURMOUNT-MMO will adjudicate whether the cardiovascular signal extends to a population where weight loss, rather than glycemic control, is the dominant treatment effect. It will complete the cardiovascular evidence base for the currently highest-efficacy approved agent.

### 11.3 What lifecycle thinking means for the field

Three implications follow: First, reframing these agents as chronic-disease pharmacotherapy requires that the trials, prescribing pathways, payer mechanisms, and clinical training stop treating the 72-week pivotal trial as the relevant unit of analysis. The clinical lifecycle is decades, and the trial-evidence-to-clinical-practice translation gap is largest in the stages the pivotal trials do not cover: maintenance, drug holidays, planned tapering, restart, agent switching, post-discontinuation cardiometabolic management, and the system-level questions of access and supply.

Second, treatment-discontinuation outcomes should be standardized endpoints in pivotal trials going forward. The cardiometabolic trajectory after discontinuation is the lifecycle’s most informative event, and the absence of pre-specified post-discontinuation outcomes has substantially limited the strength of the evidence supporting the chronic-pharmacotherapy framing.

Third, implementation effectiveness, not trial efficacy, is the relevant performance metric. The 8% to 9% real-world weight loss in community EHR cohorts (noted earlier under real-world titration) is what these agents accomplish at population scale under current conditions, and the gap between that figure and the trial figure of 15%–21% is the work to be done.

## 12 Conclusions

Two decades after exenatide reached clinical practice, the cardiometabolic incretin class has reshaped the therapeutic landscape across a remarkable range of indications. The pivotal trial evidence is now mature, and the efficacy data for both selective GLP-1 receptor agonists and dual GIP/GLP-1 receptor agonists are durable, reproducible, and clinically meaningful. The lifecycle questions remain less well characterized. The trajectory of benefit during continued therapy is reasonably well described, but the trajectory after discontinuation, the dynamics of cyclical use, the patterns of agent switching, the maintenance-and-de-escalation evidence space, and the system-level determinants of access continuity have not been synthesized into a coherent framework.

Our review has attempted that synthesis. Seven analytical positions have been advanced. First, the GIPR pharmacology debate is partly a category error, with the cellular outcome at three tissue compartments being more clinically relevant than the agonist-versus-antagonist binary. Second, the tirzepatide-versus-semaglutide differential is partly dose-equivalence-driven and partly mechanism-driven. Third, the class is best framed as chronic-disease pharmacotherapy rather than episodic-titration agents. Fourth, in the average trial-population adult, the pattern of lean-mass loss with incretin therapy is consistent with adaptive remodeling, while older adults, adults with pre-existing sarcopenia, and adults with modest baseline muscle reserve warrant stratified prescription and, where indicated, structured resistance training and adequate protein intake. Fifth, periprocedural management should be individualized by setting and titration status rather than handled with blanket withholding. Sixth, the strongest available evidence, drawn from SELECT and SUMMIT mediation and secondary analyses, is consistent with a substantial weight-independent contribution to cardiovascular benefit, though the mediation reasoning is observational and *post-hoc*, and cannot prove mechanism independent of weight. Seventh, the neuropsychiatric signal is not supported at the population level by current epidemiological evidence, while continued pharmacovigilance remains appropriate.

These positions converge on a single observation. The determinants of long-term benefit lie largely outside the efficacy envelope defined by trial endpoints. The pivotal trial literature has answered most of the questions about initiation and on-treatment trajectory and has left most of the questions about persistence, discontinuation, reinitiation, and maintenance unanswered. The research agenda developed above names the five trial designs the field requires. Five knowledge gaps warrant emphasis for the field’s next decade, namely: adequately powered randomized continuation-versus-withdrawal trials at year 2 in sustained responders, structured drug-holiday and reinitiation protocols to test whether cyclical use delivers comparable cardiometabolic benefit, agent-switching trials in first-agent non-responders, standardized post-discontinuation outcomes as prespecified endpoints in future pivotal trials, and prospective evaluation of implementation and access as determinants of population-level effect. The implementation agenda implicit throughout names the system-level changes that will determine whether this class accomplishes at population scale what it accomplishes in pivotal trials.

## References

[1] Müller TD Finan B Bloom SR D'Alessio D Drucker DJ Flatt PR . Glucagon-like peptide 1 (GLP-1). Mol Metab. (2019) 30:72–130. doi: 10.1016/j.molmet.2019.09.010 31767182 PMC6812410

[2] Jastreboff AM Aronne LJ Ahmad NN Wharton S Connery L Alves B . Tirzepatide once weekly for the treatment of obesity. New Engl J Medicine. (2022) 387:205–16. doi: 10.1056/NEJMoa2206038 35658024

[3] Borlaug BA Zile MR Kramer CM Baum SJ Hurt K Litwin SE . Effects of tirzepatide on circulatory overload and end-organ damage in heart failure with preserved ejection fraction and obesity: a secondary analysis of the SUMMIT trial. Nat Medicine. (2025) 31:544–51. doi: 10.1038/s41591-024-03374-z 39551891 PMC11835708

[4] Malhotra A Grunstein RR Fietze I Weaver TE Redline S Azarbarzin A . Tirzepatide for the treatment of obstructive sleep apnea and obesity. New Engl J Medicine. (2024) 391:1193–205. doi: 10.1056/NEJMoa2404881 38912654 PMC11598664

[5] McGuire DK Marx N Mulvagh SL Deanfield JE Inzucchi SE Pop-Busui R . Oral semaglutide and cardiovascular outcomes in high-risk type 2 diabetes. New Engl J Medicine. (2025) 392:2001–12. doi: 10.1056/NEJMoa2501006 40162642

[6] Lincoff AM Brown-Frandsen K Colhoun HM Deanfield J Emerson SS Esbjerg S . Semaglutide and cardiovascular outcomes in obesity without diabetes. New Engl J Medicine. (2023) 389:2221–32. doi: 10.1056/NEJMoa2307563 37952131

[7] Perkovic V Tuttle KR Rossing P Mahaffey KW Mann JFE Bakris G . Effects of semaglutide on chronic kidney disease in patients with type 2 diabetes. New Engl J Medicine. (2024) 391:109–21. doi: 10.1056/NEJMoa2403347 38785209

[8] Loomba R Hartman ML Lawitz EJ Vuppalanchi R Boursier J Bugianesi E . Tirzepatide for metabolic dysfunction-associated steatohepatitis with liver fibrosis. New Engl J Medicine. (2024) 391:299–310. doi: 10.1056/NEJMoa2401943 38856224

[9] Willard FS Douros JD Gabe MBN Showalter AD Wainscott DB Suter TM . Tirzepatide is an imbalanced and biased dual GIP and GLP-1 receptor agonist. JCI Insight. (2020) 5:e140532. doi: 10.1172/jci.insight.140532 32730231 PMC7526454

[10] Sarma S Palcu P . Weight loss between glucagon-like peptide-1 receptor agonists and bariatric surgery in adults with obesity: a systematic review and meta-analysis. Obesity. (2022) 30:2111–21. doi: 10.1002/oby.23563 36321278

[11] Gleason PP Urick BY Marshall LZ Friedlander N Qiu Y Leslie RS . Real-world persistence and adherence to glucagon-like peptide-1 receptor agonists among obese commercially insured adults without diabetes. J Managed Care Specialty Pharmacy. (2024) 30:860–7. doi: 10.18553/jmcp.2024.23332 38717042 PMC11293763

[12] Rodriguez PJ Zhang V Gratzl S Do D Goodwin Cartwright BM Baker C . Discontinuation and reinitiation of dual-labeled GLP-1 receptor agonists among US adults with overweight or obesity. JAMA Netw Open. (2025) 8:e2457349. doi: 10.1001/jamanetworkopen.2024.57349 39888616 PMC11786232

[13] Gasoyan H Butsch WS Schulte R Casacchia NJ Le P Boyer CB . Changes in weight and glycemic control following obesity treatment with semaglutide or tirzepatide by discontinuation status. Obesity. (2025) 33:1657–67. doi: 10.1002/oby.24331 40491239 PMC12381620

[14] Wilding JPH Batterham RL Davies M Van Gaal LF Kandler K Konakli K . Weight regain and cardiometabolic effects after withdrawal of semaglutide: the STEP 1 trial extension. Diabetes Obesity Metab. (2022) 24:1553–64. doi: 10.1111/dom.14725 35441470 PMC9542252

[15] Berg S Stickle H Rose SJ Nemec EC . Discontinuing glucagon-like peptide-1 receptor agonists and body habitus: a systematic review and meta-analysis. Obesity Rev. (2025) 26:e13929. doi: 10.1111/obr.13929 40186344

[16] Aronne LJ Sattar N Horn DB Bays HE Wharton S Lin WY . Continued treatment with tirzepatide for maintenance of weight reduction in adults with obesity: the SURMOUNT-4 randomized clinical trial. JAMA. (2024) 331:38–48. doi: 10.1001/jama.2023.24945 38078870 PMC10714284

[17] Piccini S Favacchio G Panico C Morenghi E Folli F Mazziotti G . Time-dependent effect of GLP-1 receptor agonists on cardiovascular benefits: a real-world study. Cardiovasc Diabetol. (2023) 22:69. doi: 10.1186/s12933-023-01800-z 36966321 PMC10039680

[18] Gasoyan H Butsch WS Casacchia NJ Schulte R Criswell V Fox J . Reasons for discontinuation of obesity pharmacotherapy with semaglutide or tirzepatide in clinical practice. Obesity. (2025) 33:2296–303. doi: 10.1002/oby.70058 41039650 PMC12636059

[19] Ulrich FS Napoli N Nielsen MF Burden AM . Real-world persistence and dose titration of GLP-1 receptor agonists in type 2 diabetes: a UK population-based cohort study by obesity and cardiovascular disease status. Diabetes Obesity Metab. (2026) 28:3386–95. doi: 10.1111/dom.70535 41703773 PMC12992168

[20] Ashruf OS Sontz EJ Akbar H Orozco Z Khan IA Mushtaq A . Disparities in access of GLP-1 receptor agonists in cancer patients with obesity or type 2 diabetes: a cascade-of-care analysis across the semaglutide shortage timeline. JCO Oncol Pract. (2025) 21:193. doi: 10.1200/OP.2025.21.10_suppl.193

[21] Aronne LJ Horn DB le Roux CW Ho W Falcon BL Gomez Valderas E . Tirzepatide as compared with semaglutide for the treatment of obesity. New Engl J Medicine. (2025) 393:26–36. doi: 10.1056/NEJMoa2416394 40353578

[22] Lam CSP Rodriguez A Aminian A Ferrannini E Heerspink HJL Jastreboff AM . Tirzepatide for reduction of morbidity and mortality in adults with obesity: rationale and design of the SURMOUNT-MMO trial. Obesity. (2025) 33:1645–56. doi: 10.1002/oby.24332 40545827

[23] Weghuber D Barrett T Barrientos-Pérez M Gies I Hesse D Jeppesen OK . Once-weekly semaglutide in adolescents with obesity. New Engl J Medicine. (2022) 387:2245–57. doi: 10.1056/NEJMoa2208601 36322838 PMC9997064

[24] Bjerre Knudsen L Madsen LW Andersen S Almholt K de Boer AS Drucker DJ . Glucagon-like peptide-1 receptor agonists activate rodent thyroid C-cells causing calcitonin release and C-cell proliferation. Endocrinology. (2010) 151:1473–86. doi: 10.1210/en.2009-1272 20203154

[25] Storgaard H Cold F Gluud LL Vilsbøll T Knop FK . Glucagon-like peptide-1 receptor agonists and risk of acute pancreatitis in patients with type 2 diabetes. Diabetes Obesity Metab. (2017) 19:906–8. doi: 10.1111/dom.12885 28105738

[26] He L Wang J Ping F Yang N Huang J Li Y . Association of glucagon-like peptide-1 receptor agonist use with risk of gallbladder and biliary diseases: a systematic review and meta-analysis of randomized clinical trials. JAMA Internal Medicine. (2022) 182:513–9. doi: 10.1001/jamainternmed.2022.0338 35344001 PMC8961394

[27] Hathaway JT Shah MP Hathaway DB Zekavat SM Krasniqi D Gittinger JW Jr . Risk of nonarteritic anterior ischemic optic neuropathy in patients prescribed semaglutide. JAMA Ophthalmol. (2024) 142:732–9. doi: 10.1001/jamaophthalmol.2024.2296 38958939 PMC11223051

[28] Hurtado I Robles C Peiró S García-Sempere A Sanfélix-Gimeno G . Association of glucagon-like peptide-1 receptor agonists with suicidal ideation and self-injury in individuals with diabetes and obesity: a propensity-weighted, population-based cohort study. Diabetologia. (2024) 67:2471–80. doi: 10.1007/s00125-024-06243-z 39103719 PMC11519213

[29] Xie Y Choi T Al-Aly Z . Mapping the effectiveness and risks of GLP-1 receptor agonists. Nat Medicine. (2025) 31:951–62. doi: 10.1038/s41591-024-03412-w 39833406

[30] Jalleh RJ Talley NJ Horowitz M Nauck MA . The science of safety: adverse effects of GLP-1 receptor agonists as glucose-lowering and obesity medications. J Clin Investigation. (2026) 136:e194740. doi: 10.1172/JCI194740 41697736 PMC12904723

[31] Nauck MA Müller TD . Incretin hormones and type 2 diabetes. Diabetologia. (2023) 66:1780–95. doi: 10.1007/s00125-023-05956-x 37430117 PMC10474001

[32] Drucker DJ Nauck MA . The incretin system: glucagon-like peptide-1 receptor agonists and dipeptidyl peptidase-4 inhibitors in type 2 diabetes. Lancet. (2006) 368:1696–705. doi: 10.1016/S0140-6736(06)69705-5 17098089

[33] Müller TD Adriaenssens A Ahrén B Blüher M Birkenfeld AL Campbell JE . Glucose-dependent insulinotropic polypeptide (GIP). Mol Metab. (2025) 95:102118. doi: 10.1016/j.molmet.2025.102118 40024571 PMC11931254

[34] Holst JJ Rosenkilde MM . GIP as a therapeutic target in diabetes and obesity: insight from incretin co-agonists. J Clin Endocrinol Metab. (2020) 105:e2710–6. doi: 10.1210/clinem/dgaa327 32459834 PMC7308078

[35] Martin CK Carmichael OT Carnell S Considine RV Kareken DA Dydak U . Tirzepatide on ingestive behavior in adults with overweight or obesity: a randomized 6-week phase 1 trial. Nat Medicine. (2025) 31:3141–50. doi: 10.1038/s41591-025-03774-9 40555748 PMC12443625

[36] Urva S Coskun T Loghin C Cui X Beebe E O'Farrell L . The novel dual glucose-dependent insulinotropic polypeptide and glucagon-like peptide-1 (GLP-1) receptor agonist tirzepatide transiently delays gastric emptying similarly to selective long-acting GLP-1 receptor agonists. Diabetes Obesity Metab. (2020) 22:1886–91. doi: 10.1111/dom.14110 32519795 PMC7539915

[37] Jalleh RJ Marathe CS Rayner CK Jones KL Umapathysivam MM Wu T . Physiology and pharmacology of effects of GLP-1-based therapies on gastric, biliary and intestinal motility. Endocrinology. (2025) 166:bqae155. doi: 10.1210/endocr/bqae155 39568409 PMC11630531

[38] Manchanda Y Bitsi S Chen S Broichhagen J Bernardino de la Serna J Jones B . Enhanced endosomal signaling and desensitization of GLP-1R vs GIPR in pancreatic beta cells. Endocrinology. (2023) 164:bqad028. doi: 10.1210/endocr/bqad028 36774542 PMC10016038

[39] Sattar N Verma S Sourij H Wilson J Stefanski A Zhang X . Abstract 4139912: Inflammatory biomarkers in people treated with tirzepatide living with overweight or obesity, without and with T2D: a post-hoc analysis from SURMOUNT-1 and SURMOUNT-2. Circulation. (2024) 150:A4139912. doi: 10.1161/circ.150.suppl_1.4139912

[40] Ussher JR Drucker DJ . Glucagon-like peptide 1 receptor agonists: cardiovascular benefits and mechanisms of action. Nat Rev Cardiol. (2023) 20:463–74. doi: 10.1038/s41569-023-00849-3 36977782

[41] Neeland IJ Marso SP Ayers CR Lewis B Oslica R Francis W . Effects of liraglutide on visceral and ectopic fat in adults with overweight and obesity at high cardiovascular risk: a randomised, double-blind, placebo-controlled, clinical trial. Lancet Diabetes Endocrinol. (2021) 9:595–605. doi: 10.1016/S2213-8587(21)00179-0 34358471

[42] Zhao F Zhou Q Cong Z Hang K Zou X Zhang C . Structural insights into multiplexed pharmacological actions of tirzepatide and peptide 20 at the GIP, GLP-1 or glucagon receptors. Nat Communications. (2022) 13:1057. doi: 10.1038/s41467-022-28683-0 35217653 PMC8881610

[43] El K Douros JD Willard FS Novikoff A Sargsyan A Perez-Tilve D . The incretin co-agonist tirzepatide requires GIPR for hormone secretion from human islets. Nat Metab. (2023) 5:945–54. doi: 10.1038/s42255-023-00811-0 37277609 PMC10290954

[44] Campbell JE . Targeting the GIPR for obesity: to agonize or antagonize? Potential mechanisms. Mol Metab. (2021) 46:101139. doi: 10.1016/j.molmet.2020.101139 33290902 PMC8085569

[45] Killion EA Chen M Falsey JR Sivits G Hager T Atangan L . Chronic glucose-dependent insulinotropic polypeptide receptor (GIPR) agonism desensitizes adipocyte GIPR activity mimicking functional GIPR antagonism. Nat Communications. (2020) 11:4981. doi: 10.1038/s41467-020-18751-8 33020469 PMC7536395

[46] Yu X Chen S Funcke JB Straub LG Pirro V Emont MP . The GIP receptor activates futile calcium cycling in white adipose tissue to increase energy expenditure and drive weight loss in mice. Cell Metab. (2025) 37:187–204.e7. doi: 10.1016/j.cmet.2024.11.003 39642881 PMC11711001

[47] Véniant MM Lu SC Atangan L Komorowski R Stanislaus S Cheng Y . A GIPR antagonist conjugated to GLP-1 analogues promotes weight loss with improved metabolic parameters in preclinical and phase 1 settings. Nat Metab. (2024) 6:290–303. doi: 10.1038/s42255-023-00966-w 38316982 PMC10896721

[48] Jastreboff AM Kaplan LM Frías JP Wu Q Du Y Gurbuz S . Triple-hormone-receptor agonist retatrutide for obesity - a phase 2 trial. New Engl J Medicine. (2023) 389:514–26. doi: 10.1056/NEJMoa2301972 37366315

[49] Baggio LL Drucker DJ . Glucagon-like peptide-1 receptor co-agonists for treating metabolic disease. Mol Metab. (2021) 46:101090. doi: 10.1016/j.molmet.2020.101090 32987188 PMC8085566

[50] Jalleh RJ Plummer MP Marathe CS Umapathysivam MM Quast DR Rayner CK . Clinical consequences of delayed gastric emptying with GLP-1 receptor agonists and tirzepatide. J Clin Endocrinol Metab. (2025) 110:1–15. doi: 10.1210/clinem/dgae719 39418085 PMC11651700

[51] Wharton S Freitas P Hjelmesæth J Kabisch M Kandler K Lingvay I . Once-weekly semaglutide 7.2 mg in adults with obesity (STEP UP): a randomised, controlled, phase 3b trial. Lancet Diabetes Endocrinol. (2025) 13:949–63. doi: 10.1016/S2213-8587(25)00226-8 40961952

[52] Samms RJ Christe ME Collins KAL Pirro V Droz BA Holland AK . GIPR agonism mediates weight-independent insulin sensitization by tirzepatide in obese mice. J Clin Investigation. (2021) 131:e146353. doi: 10.1172/JCI146353 34003802 PMC8203452

[53] Thomas MK Nikooienejad A Bray R Cui X Wilson J Duffin K . Dual GIP and GLP-1 receptor agonist tirzepatide improves beta-cell function and insulin sensitivity in type 2 diabetes. J Clin Endocrinol Metab. (2021) 106:388–96. doi: 10.1210/clinem/dgaa863 33236115 PMC7823251

[54] Rosenstock J Wysham C Frías JP Kaneko S Lee CJ Fernández Landó L . Efficacy and safety of a novel dual GIP and GLP-1 receptor agonist tirzepatide in patients with type 2 diabetes (SURPASS-1): a double-blind, randomised, phase 3 trial. Lancet. (2021) 398:143–55. doi: 10.1016/S0140-6736(21)01324-6 34186022

[55] Ludvik B Giorgino F Jódar E Frias JP Fernández Landó L Brown K . Once-weekly tirzepatide versus once-daily insulin degludec as add-on to metformin with or without SGLT2 inhibitors in patients with type 2 diabetes (SURPASS-3): a randomised, open-label, parallel-group, phase 3 trial. Lancet. (2021) 398:583–98. doi: 10.1016/S0140-6736(21)01443-4 34370970

[56] Del Prato S Kahn SE Pavo I Weerakkody GJ Yang Z Doupis J . Tirzepatide versus insulin glargine in type 2 diabetes and increased cardiovascular risk (SURPASS-4): a randomised, open-label, parallel-group, multicentre, phase 3 trial. Lancet. (2021) 398:1811–24. doi: 10.1016/S0140-6736(21)02188-7 34672967

[57] Frías JP Davies MJ Rosenstock J Pérez Manghi FC Fernández Landó L Bergman BK . Tirzepatide versus semaglutide once weekly in patients with type 2 diabetes. New Engl J Medicine. (2021) 385:503–15. doi: 10.1056/NEJMoa2107519 34170647

[58] Nauck MA D'Alessio DA . Tirzepatide, a dual GIP/GLP-1 receptor co-agonist for the treatment of type 2 diabetes with unmatched effectiveness regrading glycaemic control and body weight reduction. Cardiovasc Diabetol. (2022) 21:169. doi: 10.1186/s12933-022-01604-7 36050763 PMC9438179

[59] Marso SP Daniels GH Brown-Frandsen K Kristensen P Mann JFE Nauck MA . Liraglutide and cardiovascular outcomes in type 2 diabetes. New Engl J Medicine. (2016) 375:311–22. doi: 10.1056/NEJMoa1603827 27295427 PMC4985288

[60] Marso SP Bain SC Consoli A Eliaschewitz FG Jódar E Leiter LA . Semaglutide and cardiovascular outcomes in patients with type 2 diabetes. New Engl J Medicine. (2016) 375:1834–44. doi: 10.1056/NEJMoa1607141 27633186

[61] Gerstein HC Colhoun HM Dagenais GR Diaz R Lakshmanan M Pais P . Dulaglutide and cardiovascular outcomes in type 2 diabetes (REWIND): a double-blind, randomised placebo-controlled trial. Lancet. (2019) 394:121–30. doi: 10.1016/S0140-6736(19)31149-3 31189511

[62] Gerstein HC Sattar N Rosenstock J Ramasundarahettige C Pratley R Lopes RD . Cardiovascular and renal outcomes with efpeglenatide in type 2 diabetes. New Engl J Medicine. (2021) 385:896–907. doi: 10.1056/NEJMoa2108269 34215025

[63] Sattar N Lee MMY Kristensen SL Branch KRH Del Prato S Khurmi NS . Cardiovascular, mortality, and kidney outcomes with GLP-1 receptor agonists in patients with type 2 diabetes: a systematic review and meta-analysis of randomised trials. Lancet Diabetes Endocrinol. (2021) 9:653–62. doi: 10.1016/S2213-8587(21)00203-5 34425083

[64] Kristensen SL Rørth R Jhund PS Docherty KF Sattar N Preiss D . Cardiovascular, mortality, and kidney outcomes with GLP-1 receptor agonists in patients with type 2 diabetes: a systematic review and meta-analysis of cardiovascular outcome trials. Lancet Diabetes Endocrinol. (2019) 7:776–85. doi: 10.1016/S2213-8587(19)30249-9 31422062

[65] Pfeffer MA Claggett B Diaz R Dickstein K Gerstein HC Køber LV . Lixisenatide in patients with type 2 diabetes and acute coronary syndrome. New Engl J Medicine. (2015) 373:2247–57. doi: 10.1056/NEJMoa1509225 26630143

[66] Holman RR Bethel MA Mentz RJ Thompson VP Lokhnygina Y Buse JB . Effects of once-weekly exenatide on cardiovascular outcomes in type 2 diabetes. New Engl J Medicine. (2017) 377:1228–39. doi: 10.1056/NEJMoa1612917 28910237 PMC9792409

[67] Jastreboff AM le Roux CW Stefanski A Aronne LJ Halpern B Wharton S . Tirzepatide for obesity treatment and diabetes prevention. New Engl J Medicine. (2025) 392:958–71. doi: 10.1056/NEJMoa2410819 39536238

[68] Wilding JPH Batterham RL Calanna S Davies M Van Gaal LF Lingvay I . Once-weekly semaglutide in adults with overweight or obesity. New Engl J Medicine. (2021) 384:989–1002. doi: 10.1056/NEJMoa2032183 33567185

[69] Pi-Sunyer X Astrup A Fujioka K Greenway F Halpern A Krempf M . A randomized, controlled trial of 3.0 mg of liraglutide in weight management. New Engl J Med. (2015) 373:11–22. doi: 10.1056/NEJMoa1411892 26132939

[70] Garvey WT Frias JP Jastreboff AM le Roux CW Sattar N Aizenberg D . Tirzepatide once weekly for the treatment of obesity in people with type 2 diabetes (SURMOUNT-2): a double-blind, randomised, multicentre, placebo-controlled, phase 3 trial. Lancet. (2023) 402:613–26. doi: 10.1016/S0140-6736(23)01200-X 37385275

[71] Davies M Færch L Jeppesen OK Pakseresht A Pedersen SD Perreault L . Semaglutide 2.4 mg once a week in adults with overweight or obesity, and type 2 diabetes (STEP 2): a randomised, double-blind, double-dummy, placebo-controlled, phase 3 trial. Lancet. (2021) 397:971–84. doi: 10.1016/S0140-6736(21)00213-0 33667417

[72] Kosiborod MN Abildstrøm SZ Borlaug BA Butler J Rasmussen S Davies M . Semaglutide in patients with heart failure with preserved ejection fraction and obesity. New Engl J Medicine. (2023) 389:1069–84. doi: 10.1056/NEJMoa2306963 37622681

[73] Verma S Butler J Borlaug BA Davies M Kitzman DW Shah SJ . Efficacy of semaglutide by sex in obesity-related heart failure with preserved ejection fraction: STEP-HFpEF trials. J Am Coll Cardiol. (2024) 84:773–85. doi: 10.1016/j.jacc.2024.06.001 38913003

[74] Kosiborod MN Verma S Borlaug BA Butler J Davies MJ Jensen TJ . Effects of semaglutide on symptoms, function, and quality of life in patients with heart failure with preserved ejection fraction and obesity: a prespecified analysis of the STEP-HFpEF trial. Circulation. (2024) 149:204–16. doi: 10.1161/CIRCULATIONAHA.123.067505 37952180 PMC10782938

[75] Packer M Zile MR Kramer CM Baum SJ Litwin SE Menon V . Tirzepatide for heart failure with preserved ejection fraction and obesity. New Engl J Medicine. (2025) 392:427–37. doi: 10.1056/NEJMoa2410027 39555826

[76] Deanfield J Verma S Scirica BM Kahn SE Emerson SS Ryan D . Semaglutide and cardiovascular outcomes in patients with obesity and prevalent heart failure: a prespecified analysis of the SELECT trial. Lancet. (2024) 404:773–86. doi: 10.1016/S0140-6736(24)01498-3 39181597

[77] Malhotra A Grunstein R Azarbarzin A Sands S Somers VK Aronne LJ . Tirzepatide on obstructive sleep apnea-related cardiometabolic risk: secondary outcomes of the SURMOUNT-OSA randomized trial. Nat Medicine. (2026) 32:653–9. doi: 10.1038/s41591-025-04071-1 41540105 PMC12920140

[78] Blackman A Foster GD Zammit G Rosenberg R Aronne L Wadden T . Effect of liraglutide 3.0 mg in individuals with obesity and moderate or severe obstructive sleep apnea: the SCALE Sleep Apnea randomized clinical trial. Int J Obesity. (2016) 40:1310–9. doi: 10.1038/ijo.2016.52 27005405 PMC4973216

[79] Newsome PN Buchholtz K Cusi K Linder M Okanoue T Ratziu V . A placebo-controlled trial of subcutaneous semaglutide in nonalcoholic steatohepatitis. New Engl J Medicine. (2021) 384:1113–24. doi: 10.1056/NEJMoa2028395 33185364

[80] Loomba R Abdelmalek MF Armstrong MJ Jara M Kjær MS Krarup N . Semaglutide 2.4 mg once weekly in patients with non-alcoholic steatohepatitis-related cirrhosis: a randomised, placebo-controlled phase 2 trial. Lancet Gastroenterol Hepatol. (2023) 8:511–22. doi: 10.1016/S2468-1253(23)00068-7 36934740 PMC10792518

[81] Sanyal AJ Kaplan LM Frias JP Brouwers B Wu Q Thomas MK . Triple hormone receptor agonist retatrutide for metabolic dysfunction-associated steatotic liver disease: a randomized phase 2a trial. Nat Medicine. (2024) 30:2037–48. doi: 10.1038/s41591-024-03018-2 38858523 PMC11271400

[82] Sanyal AJ Bedossa P Fraessdorf M Neff GW Lawitz E Bugianesi E . A phase 2 randomized trial of survodutide in MASH and fibrosis. New Engl J Medicine. (2024) 391:311–9. doi: 10.1056/NEJMoa2401755 38847460

[83] Harrison SA Browne SK Suschak JJ Tomah S Gutierrez JA Yang J . Effect of pemvidutide, a GLP-1/glucagon dual receptor agonist, on MASLD: a randomized, double-blind, placebo-controlled study. J Hepatol. (2025) 82:7–17. doi: 10.1016/j.jhep.2024.07.006 39002641

[84] Harrison SA Bedossa P Guy CD Schattenberg JM Loomba R Taub R . A phase 3, randomized, controlled trial of resmetirom in NASH with liver fibrosis. New Engl J Medicine. (2024) 390:497–509. doi: 10.1056/NEJMoa2309000 38324483

[85] Marx N Deanfield JE Mann JFE Arechavaleta R Bain SC Bajaj HS . Oral semaglutide and cardiovascular outcomes in people with type 2 diabetes, according to SGLT2i use: prespecified analyses of the SOUL randomized trial. Circulation. (2025) 151:1639–50. doi: 10.1161/CIRCULATIONAHA.125.074545 40156843 PMC12144549

[86] Colhoun HM Lingvay I Brown PM Deanfield J Brown-Frandsen K Kahn SE . Long-term kidney outcomes of semaglutide in obesity and cardiovascular disease in the SELECT trial. Nat Medicine. (2024) 30:2058–66. doi: 10.1038/s41591-024-03015-5 38796653 PMC11271413

[87] Mann JFE Rossing P Bakris G Belmar N Bosch-Traberg H Busch R . Effects of semaglutide with and without concomitant SGLT2 inhibitor use in participants with type 2 diabetes and chronic kidney disease in the FLOW trial. Nat Medicine. (2024) 30:2849–56. doi: 10.1038/s41591-024-03133-0 38914124 PMC11485243

[88] American Diabetes Association Professional Practice Committee . 8. Obesity and weight management for the prevention and treatment of type 2 diabetes: Standards of Care in Diabetes-2024. Diabetes Care. (2024) 47:S145–57. doi: 10.2337/dc24-S008 38078578 PMC10725806

[89] American Diabetes Association Professional Practice Committee . 9. Pharmacologic approaches to glycemic treatment: Standards of Care in Diabetes-2024. Diabetes Care. (2024) 47:S158–78. doi: 10.2337/dc24-S009 38078590 PMC10725810

[90] American Diabetes Association Professional Practice Committee . 8. Obesity and weight management for the prevention and treatment of type 2 diabetes: Standards of Care in Diabetes-2025. Diabetes Care. (2025) 48:S167–80. doi: 10.2337/dc25-S008 39651976 PMC11635032

[91] Kidney Disease: Improving Global Outcomes (KDIGO) Diabetes Work Group . KDIGO 2022 clinical practice guideline for diabetes management in chronic kidney disease. Kidney Int. (2022) 102:S1–S127. doi: 10.1016/j.kint.2022.06.008 36272764

[92] de Boer IH Khunti K Sadusky T Tuttle KR Neumiller JJ Rhee CM . Diabetes management in chronic kidney disease: a consensus report by the American Diabetes Association (ADA) and Kidney Disease: Improving Global Outcomes (KDIGO). Kidney Int. (2022) 102:974–89. doi: 10.1016/j.kint.2022.08.012 36202661

[93] Kidney Disease: Improving Global Outcomes (KDIGO) CKD Work Group . KDIGO 2024 clinical practice guideline for the evaluation and management of chronic kidney disease. Kidney Int. (2024) 105:S117–314. doi: 10.1016/j.kint.2023.10.018 38490803

[94] Wolff Sagy Y Lavie G Ramot N Battat E Arbel R Reges O . Effectiveness of bariatric metabolic surgery versus glucagon-like peptide-1 receptor agonists for prevention of congestive heart failure. Nat Medicine. (2024) 30:2337–42. doi: 10.1038/s41591-024-03052-0 38749475

[95] Rubino DM Pedersen SD Connery L Cao D Chigutsa F Stefanski A . Gastrointestinal tolerability and weight reduction associated with tirzepatide in adults with obesity or overweight with and without type 2 diabetes in the SURMOUNT-1 to -4 trials. Diabetes Obesity Metab. (2025) 27:1826–35. doi: 10.1111/dom.16176 39789843 PMC11885085

[96] Samuels JM Ye F Irlmeier R Silver H Srivastava G Spann M . Real-world titration, persistence & weight loss of semaglutide and tirzepatide in an academic obesity clinic. Diabetes Obesity Metab. (2025) 27:6200–9. doi: 10.1111/dom.70004 40762026 PMC12515774

[97] Bensignor MO Bramante CT Bomberg EM Fox CK Hale PM Kelly AS . Evaluating potential predictors of weight loss response to liraglutide in adolescents with obesity: a *post hoc* analysis of the randomized, placebo-controlled SCALE teens trial. Pediatr Obesity. (2023) 18:e13061. doi: 10.1111/ijpo.13061 37264767 PMC10926323

[98] Rodriguez PJ Goodwin Cartwright BM Gratzl S Brar R Baker C Gluckman TJ . Semaglutide vs tirzepatide for weight loss in adults with overweight or obesity. JAMA Internal Medicine. (2024) 184:1056–64. doi: 10.1001/jamainternmed.2024.2525 38976257 PMC11231910

[99] Garvey WT Batterham RL Bhatta M Buscemi S Christensen LN Frias JP . Two-year effects of semaglutide in adults with overweight or obesity: the STEP 5 trial. Nat Medicine. (2022) 28:2083–91. doi: 10.1038/s41591-022-02026-4 36216945 PMC9556320

[100] Ryan DH Lingvay I Deanfield J Kahn SE Barros E Burguera B . Long-term weight loss effects of semaglutide in obesity without diabetes in the SELECT trial. Nat Medicine. (2024) 30:2049–57. doi: 10.1038/s41591-024-02996-7 38740993 PMC11271387

[101] le Roux CW Astrup A Fujioka K Greenway F Lau DCW Van Gaal L . 3 years of liraglutide versus placebo for type 2 diabetes risk reduction and weight management in individuals with prediabetes: a randomised, double-blind trial. Lancet. (2017) 389:1399–409. doi: 10.1016/S0140-6736(17)30069-7 28237263

[102] Kanwal F Kramer JR Li L Yang YX Cao Y Yu X . GLP-1 receptor agonists and risk for cirrhosis and related complications in patients with metabolic dysfunction-associated steatotic liver disease. JAMA Internal Medicine. (2024) 184:1314–23. doi: 10.1001/jamainternmed.2024.4661 39283612 PMC11406452

[103] Look M Dunn JP Kushner RF Cao D Harris C Gibble TH . Body composition changes during weight reduction with tirzepatide in the SURMOUNT-1 study of adults with obesity or overweight. Diabetes Obesity Metab. (2025) 27:2720–9. doi: 10.1111/dom.16275 39996356 PMC11965027

[104] Sattar N Neeland IJ Dahlqvist Leinhard O Fernández Landó L Bray R Linge J . Tirzepatide and muscle composition changes in people with type 2 diabetes (SURPASS-3 MRI): a post-hoc analysis of a randomised, open-label, parallel-group, phase 3 trial. Lancet Diabetes Endocrinol. (2025) 13:482–93. doi: 10.1016/S2213-8587(25)00027-0 40318682

[105] Neeland IJ Linge J Birkenfeld AL . Changes in lean body mass with glucagon-like peptide-1-based therapies and mitigation strategies. Diabetes Obesity Metab. (2024) 26:16–27. doi: 10.1111/dom.15728 38937282

[106] Batsis JA Villareal DT . Sarcopenic obesity in older adults: aetiology, epidemiology and treatment strategies. Nat Rev Endocrinol. (2018) 14:513–37. doi: 10.1038/s41574-018-0062-9 30065268 PMC6241236

[107] Ryan DH . New drugs for the treatment of obesity: do we need approaches to preserve muscle mass? Rev Endocr Metab Disord. (2025) 26:805–13. doi: 10.1007/s11154-025-09967-4 40320499 PMC12534358

[108] Melson E Ashraf U Papamargaritis D Davies MJ . What is the pipeline for future medications for obesity? Int J Obesity. (2025) 49:433–51. doi: 10.1038/s41366-024-01473-y 38302593 PMC11971045

[109] Monami M Nreu B Scatena A Cresci B Andreozzi F Sesti G . Safety issues with glucagon-like peptide-1 receptor agonists (pancreatitis, pancreatic cancer and cholelithiasis): data from randomized controlled trials. Diabetes Obesity Metab. (2017) 19:1233–41. doi: 10.1111/dom.12926 28244632

[110] Ko A Chang YC Bahar F Wang TH Xanthavanij N Yu CC . Risk for cancer with glucagon-like peptide-1 receptor agonists and dual agonists: a systematic review and meta-analysis. Ann Internal Medicine. (2026) 179:216–29. doi: 10.7326/ANNALS-25-02237 41359966

[111] Morales DR Bu F Viernes B DuVall SL Matheny ME Simon KR . Risk of thyroid tumors with GLP-1 receptor agonists: a retrospective cohort study. Diabetes Care. (2025) 48:1386–94. doi: 10.2337/dc25-0154 40465422 PMC12281980

[112] Kelly CA Sipos JA . Approach to the patient with thyroid nodules: considering GLP-1 receptor agonists. J Clin Endocrinol Metab. (2025) 110:e2080–7. doi: 10.1210/clinem/dgae722 39400117

[113] Cai CX Hribar M Baxter S Goetz K Swaminathan SS Flowers A . Semaglutide and nonarteritic anterior ischemic optic neuropathy. JAMA Ophthalmol. (2025) 143:304–14. doi: 10.1001/jamaophthalmol.2024.6555 39976940 PMC11843465

[114] Simonsen E Lund LC Ernst MT Hjellvik V Hegedüs L Hamann S . Use of semaglutide and risk of non-arteritic anterior ischemic optic neuropathy: a Danish-Norwegian cohort study. Diabetes Obesity Metab. (2025) 27:3094–103. doi: 10.1111/dom.16316 40098249 PMC12046482

[115] Wang L Volkow ND Kaelber DC Xu R . Semaglutide or tirzepatide and optic nerve and visual pathway disorders in type 2 diabetes. JAMA Netw Open. (2025) 8:e2526327. doi: 10.1001/jamanetworkopen.2025.26327 40788646 PMC12340659

[116] Lassen MCH Johansen ND Modin D Catarig AM Vistisen BK Amadid H . Adherence to glucagon-like peptide-1 receptor agonist treatment in type 2 diabetes mellitus: a nationwide registry study. Diabetes Obesity Metab. (2024) 26:5239–50. doi: 10.1111/dom.15872 39215626

[117] Do D Lee T Peasah SK Good CB Inneh A Patel U . GLP-1 receptor agonist discontinuation among patients with obesity and/or type 2 diabetes. JAMA Netw Open. (2024) 7:e2413172. doi: 10.1001/jamanetworkopen.2024.13172 38787563 PMC11127113

[118] Rubino D Abrahamsson N Davies M Hesse D Greenway FL Jensen C . Effect of continued weekly subcutaneous semaglutide vs placebo on weight loss maintenance in adults with overweight or obesity: the STEP 4 randomized clinical trial. JAMA. (2021) 325:1414–25. doi: 10.1001/jama.2021.3224 33755728 PMC7988425

[119] Wadden TA Hollander P Klein S Niswender K Woo V Hale PM . Weight maintenance and additional weight loss with liraglutide after low-calorie-diet-induced weight loss: the SCALE Maintenance randomized study. Int J Obesity. (2013) 37:1443–51. doi: 10.1038/ijo.2013.120 23812094

[120] Sørensen KK Yazdanfard PDW Zareini B Pedersen-Bjergaard U Kosjerina V Andersen MP . Real-world cardiovascular effectiveness of sustained glucagon-like peptide 1 GLP-1 receptor agonist usage in type 2 diabetes. Cardiovasc Diabetol. (2025) 24:385. doi: 10.1186/s12933-025-02915-1 41053738 PMC12502128

[121] Moiz A Filion KB Tsoukas MA Yu OHY Peters TM Eisenberg MJ . The expanding role of GLP-1 receptor agonists: a narrative review of current evidence and future directions. EClinicalMedicine. (2025) 86:103363. doi: 10.1016/j.eclinm.2025.103363 40727007 PMC12303005

[122] Ushakumari DS Sladen RN . ASA consensus-based guidance on preoperative management of patients on glucagon-like peptide-1 receptor agonists. Anesthesiology. (2024) 140:346–8. doi: 10.1097/ALN.0000000000004776 37982170

[123] Castrillon J Rele S Sumithran P Hii M Thuraisingam S Spelman T . Association between glucagon-like peptide-1 receptor agonist use and peri-operative pulmonary aspiration: a systematic review and meta-analysis. Anaesthesia. (2025) 80:846–58. doi: 10.1111/anae.16601 40230298 PMC12171790

[124] Facciorusso A Ramai D Dhar J Samanta J Chandan S Gkolfakis P . Effects of glucagon-like peptide-1 receptor agonists on upper gastrointestinal endoscopy: a meta-analysis. Clin Gastroenterol Hepatol. (2025) 23:715–725.e3. doi: 10.1016/j.cgh.2024.07.021 39142543

[125] Pai SL Nimma SR Beam WB VanderWielen BA Kalagara HK Bettini LM . Assessment of gastric content using gastric ultrasound in patients on glucagon-like peptide-1 receptor agonists before anesthesia. Anesthesia Analgesia. (2026) 142:640–9. doi: 10.1213/ANE.0000000000007764 41032460

[126] Kindel TL Wang AY Wadhwa A Schulman AR Sharaiha RZ Kroh M . Multi-society clinical practice guidance for the safe use of glucagon-like peptide-1 receptor agonists in the perioperative period. Surg Endoscopy. (2025) 39:180–3. doi: 10.1007/s00464-024-11263-2 39370500 PMC11666732

[127] Fox CK Barrientos-Pérez M Bomberg EM Dcruz J Gies I Harder-Lauridsen NM . Liraglutide for children 6 to <12 years of age with obesity - a randomized trial. New Engl J Med. (2025) 392:555–65. doi: 10.1056/NEJMoa2407379 39258838

[128] Tang M Shi L Guan D Winberg D Tang T Shao H . Long-term cost-effectiveness of tirzepatide for individuals with type 2 diabetes and comorbid obesity. Diabetes Obesity Metab. (2025) 27:4288–99. doi: 10.1111/dom.16466 40452343

[129] Wharton S Aronne LJ Stefanski A Alfaris NF Ciudin A Yokote K . Orforglipron, an oral small-molecule GLP-1 receptor agonist for obesity treatment. New Engl J Medicine. (2025) 393:1796–806. doi: 10.1056/NEJMoa2511774 40960239

[130] Horn DB Ryan DH Kis SG Alves B Mu Y Kim SG . Orforglipron, an oral small-molecule GLP-1 receptor agonist, for the treatment of obesity in people with type 2 diabetes (ATTAIN-2): a phase 3, double-blind, randomised, multicentre, placebo-controlled trial. Lancet. (2025) 406:2927–44. doi: 10.1016/S0140-6736(25)02165-8 41275875

[131] Rosenstock J Hsia S Nevarez Ruiz L Eyde S Cox D Wu WS . Orforglipron, an oral small-molecule GLP-1 receptor agonist, in early type 2 diabetes. New Engl J Medicine. (2025) 393:1065–76. doi: 10.1056/NEJMoa2505669 40544435

[132] Knop FK Aroda VR do Vale RD Holst-Hansen T Laursen PN Rosenstock J . Oral semaglutide 50 mg taken once per day in adults with overweight or obesity (OASIS 1): a randomised, double-blind, placebo-controlled, phase 3 trial. Lancet. (2023) 402:705–19. doi: 10.1016/S0140-6736(23)01185-6 37385278

[133] Garvey WT Blüher M Osorto Contreras CK Davies MJ Winning Lehmann E Pietiläinen KH . Coadministered cagrilintide and semaglutide in adults with overweight or obesity. New Engl J Medicine. (2025) 393:635–47. doi: 10.1056/NEJMoa2502081 40544433

[134] Lawitz EJ Fraessdorf M Neff GW Schattenberg JM Noureddin M Alkhouri N . Efficacy, tolerability and pharmacokinetics of survodutide, a glucagon/glucagon-like peptide-1 receptor dual agonist, in cirrhosis. J Hepatol. (2024) 81:837–46. doi: 10.1016/j.jhep.2024.06.003 38857788

[135] Boehringer Ingelheim . LIVERAGE™: A study to test whether survodutide helps people with a liver disease called NASH/MASH who have moderate or advanced liver fibrosis. ClinicalTrials.gov. Available at: https://clinicaltrials.gov/study/NCT06632444. Identifier: NCT06632444.

[136] Aronne LJ Harris MS Roberts MS Suschak JJ Tomah S Kasper J . 262-OR: Pemvidutide, a GLP-1/glucagon dual receptor agonist, in subjects with overweight or obesity - a 48-week, placebo-controlled, phase 2 (MOMENTUM) trial. Diabetes. (2024) 73:262–OR. doi: 10.2337/db24-262-OR

[137] Ji L Gao L Jiang H Yang J Yu L Wen J . Safety and efficacy of a GLP-1 and glucagon receptor dual agonist mazdutide (IBI362) 9 mg and 10 mg in Chinese adults with overweight or obesity: a randomised, placebo-controlled, multiple-ascending-dose phase 1b trial. EClinicalMedicine. (2022) 54:101691. doi: 10.1016/j.eclinm.2022.101691 36247927 PMC9561728

[138] Nicholls SJ Pavo I Bhatt DL Buse JB Del Prato S Kahn SE . Cardiovascular outcomes with tirzepatide versus dulaglutide in type 2 diabetes. New Engl J Medicine. (2025) 393:2409–20. doi: 10.1056/NEJMoa2505928 41406444

[139] Jastreboff AM Ryan DH Bays HE Ebeling PR Mackowski MG Philipose N . Once-monthly maridebart cafraglutide for the treatment of obesity - a phase 2 trial. New Engl J Medicine. (2025) 393:843–57. doi: 10.1056/NEJMoa2504214 40549887

[140] Lieberman RA Korona-Bailey J Banaag A Furhman B Corrado R Koehlmoos TP . Semaglutide decreases risk of non-arteritic anterior ischemic optic neuroapthy in type 2 diabetic patients. Military Med. (2026) 191:e1020–5. doi: 10.1093/milmed/usaf522 41217382

[141] Sanyal AJ Newsome PN Kliers I Østergaard LH Long MT Kjær MS . Phase 3 trial of semaglutide in metabolic dysfunction-associated steatohepatitis. New Engl J Medicine. (2025) 392:2089–99. doi: 10.1056/NEJMoa2413258 40305708

[142] U.S. Food and Drug Administration . FDA approves treatment for serious liver disease known as 'MASH'. Silver Spring MD: U.S. Food and Drug Administration (2025). Available at: https://www.fda.gov/drugs/news-events-human-drugs/fda-approves-treatment-serious-liver-disease-known-mash (Accessed May 11, 2026).

[143] Novo Nordisk A/S . CagriSema demonstrated 23% weight loss in an open-label head-to-head REDEFINE 4 trial in people with obesity, the primary endpoint was not achieved. Company announcement. Bagsværd: Novo Nordisk A/S (2026). Available at: https://www.novonordisk.com/news-and-media/news-and-ir-materials/news-details.html?id=916501 (Accessed May 11, 2026).

[144] American Diabetes Association Professional Practice Committee for Diabetes . 8. Obesity and weight management for the prevention and treatment of diabetes: Standards of Care in Diabetes-2026. Diabetes Care. (2026) 49:S166–82. doi: 10.2337/dc26-S008 41358882 PMC12690172

[145] American Diabetes Association Professional Practice Committee for Diabetes . 9. Pharmacologic approaches to glycemic treatment: Standards of Care in Diabetes-2026. Diabetes Care. (2026) 49:S183–215. doi: 10.2337/dc26-S009 41358900 PMC12690185

[146] Boehringer Ingelheim . LIVERAGE™ - Cirrhosis: A study to test whether survodutide helps people with a liver disease called NASH/MASH who have cirrhosis. ClinicalTrials.gov. Available at: https://clinicaltrials.gov/study/NCT06632457. Identifier: NCT06632457.

[147] Alissou M Demangeat T Folope V Van Elslande H Lelandais H Blanchemaison J . Impact of semaglutide on fat mass, lean mass and muscle function in patients with obesity: the SEMALEAN study. Diabetes Obesity Metab. (2026) 28:112–21. doi: 10.1111/dom.70141 41068996 PMC12673431

[148] Hwang JH Laiteerapong N Huang ES Mozaffarian D Fendrick AM Kim DD . Fiscal impact of expanded Medicare coverage for GLP-1 receptor agonists to treat obesity. JAMA Health Forum. (2025) 6:e250905. doi: 10.1001/jamahealthforum.2025.0905 40279111 PMC12032556

[149] Paternò V Geraci G Piticchio T Le Moli R Burgio S Costanzo G . Mediterranean diet adherence and tirzepatide: real-world evidence on adiposity indices and insulin resistance beyond weight loss. Front Endocrinol. (2026) 16:1700894. doi: 10.3389/fendo.2025.1700894 41613958 PMC12846957

